# A Role for microRNA-155 Modulation in the Anti-HIV-1 Effects of Toll-Like Receptor 3 Stimulation in Macrophages

**DOI:** 10.1371/journal.ppat.1002937

**Published:** 2012-09-20

**Authors:** Gokul Swaminathan, Fiorella Rossi, Luz-Jeannette Sierra, Archana Gupta, Sonia Navas-Martín, Julio Martín-García

**Affiliations:** 1 Department of Microbiology and Immunology, Drexel University College of Medicine, Philadelphia, Pennsylvania, United States of America; 2 Center for Molecular Virology and Translational Neuroscience, Institute for Molecular Medicine and Infectious Disease, Drexel University College of Medicine, Philadelphia, Pennsylvania, United States of America; University of Geneva, Switzerland

## Abstract

HIV-1 infection of macrophages plays a key role in viral pathogenesis and progression to AIDS. Polyinosine-polycytidylic acid (poly(I∶C); a synthetic analog of dsRNA) and bacterial lipopolysaccharide (LPS), the ligands for Toll-like receptors (TLR) TLR3 and TLR4, respectively, are known to decrease HIV-1 infection in monocyte-derived macrophages (MDMs), but the mechanism(s) are incompletely understood. We found that poly(I∶C)- and LPS-stimulation of MDMs abrogated infection by CCR5-using, macrophage-tropic HIV-1, and by vesicular stomatitis virus glycoprotein-pseudotyped HIV-1 virions, while TLR2, TLR7 or TLR9 agonists only partially reduced infection to varying extent. Suppression of infection, or lack thereof, did not correlate with differential effects on CD4 or CCR5 expression, type I interferon induction, or production of pro-inflammatory cytokines or β-chemokines. Integrated pro-viruses were readily detected in unstimulated, TLR7- and TLR9-stimulated cells, but not in TLR3- or TLR4-stimulated MDMs, suggesting the alteration of post-entry, pre-integration event(s). Using microarray analysis and quantitative reverse transcription (RT)-PCR, we found increased microRNA (miR)-155 levels in MDMs upon TLR3/4- but not TLR7-stimulation, and a miR-155 specific inhibitor (but not a scrambled control) partially restored infectivity in poly(I∶C)-stimulated MDMs. Ectopic miR-155 expression remarkably diminished HIV-1 infection in primary MDMs and cell lines. Furthermore, poly(I∶C)-stimulation and ectopic miR-155 expression did not alter detection of early viral RT products, but both resulted in an accumulation of late RT products and in undetectable or extremely low levels of integrated pro-viruses and 2-LTR circles. Reduced mRNA and protein levels of several HIV-1 dependency factors involved in trafficking and/or nuclear import of pre-integration complexes (ADAM10, TNPO3, Nup153, LEDGF/p75) were found in poly(I∶C)-stimulated and miR-155-transfected MDMs, and a reporter assay suggested they are authentic miR-155 targets. Our findings provide evidence that miR-155 exerts an anti-HIV-1 effect by targeting several HIV-1 dependency factors involved in post-entry, pre-integration events, leading to severely diminished HIV-1 infection.

## Author Summary

The infection of macrophages by HIV-1 is a crucial event in the pathogenesis of AIDS. Toll-like receptors (TLR) are a family of receptors present in macrophages – among other cells – that detect various components of microbes and trigger host defenses. It is known that stimulation of macrophages through TLR3 or TLR4 reduces their susceptibility to HIV-1 infection, but the mechanism is not well understood. Here we show for the first time in primary human macrophages that TLR3 and TLR4 but not other TLRs induce higher levels of microRNA-155 – a key regulator of inflammatory and immune responses – and that microRNA-155 has a remarkable anti-HIV-1 effect. MicroRNAs are small, non-coding RNAs that bind to target mRNAs based on sequence complementarity, and lead to reduced protein output. We also show that the anti-HIV-1 effects of microRNA-155 seem to be mediated through targeting the mRNAs of several cellular proteins needed by the virus for trafficking and/or nuclear import of the viral DNA, which is required for integration into the host DNA and successful infection. These studies provide evidence of novel microRNA-155 targets and may serve as the basis for an innovative approach to reduce cellular susceptibility to HIV-1 infection.

## Introduction

Human immunodeficiency virus type 1 (HIV-1) infection of monocytes/macrophages plays a key role in viral pathogenesis and progression to AIDS. Macrophages contribute to early-stage viral transmission, persistence, and virus dissemination throughout the body, and accumulate replication-competent HIV-1 for prolonged periods, even in patients receiving antiretroviral treatment. With their ability to migrate into tissues, infected monocytes and macrophages are potent agents for delivery of HIV-1 to all tissues and organs, including the brain [1], [2]. Intrinsic factors have been recently suggested to play a role in a reduced susceptibility to infection of macrophages from HIV-1 controllers [3], underscoring the importance of monocyte/macrophages in the pathogenesis of AIDS.

Toll-like receptors (TLRs) are pathogen-recognition receptors that recognize integral structural components of microbes or pathogen-associated molecular patterns and play a role in the regulation of both innate and adaptive immunity, and their expression by monocytes/macrophages is of particular relevance. Stimulation of TLR-dependent pathways leads to activation of transcription factors such as nuclear factor-kappa B (NF-κB) and the expression of type I interferons (IFNs), pro-inflammatory cytokines and chemokines [4], [5]. The role of the innate immune response, and in particular TLRs, in HIV-1 infection and disease progression still remains poorly understood. The endosomal TLR7 and TLR8 recognize HIV-1 single-stranded RNA [6]–[9], but HIV-1 also seems to activate macrophages independent of TLR signaling [10]. Since TLR triggering results in immune activation, it has been suggested to contribute to HIV-1 pathogenesis [7], [11]. However, bacterial lypopolysaccharide (LPS, a TLR4 ligand) inhibits HIV-1 replication in, and infection of macrophages, and a variety of mechanisms have been proposed [12]–[18]. While stimulation through TLR2, TLR3, TLR4 or TLR5 had no or very limited effect on HIV-1 infection in human aggregate lymphocyte cultures [19], *ex vivo* HIV-1 infection of human lymphoid tissue was reduced upon TLR9 stimulation and augmented upon TLR5 stimulation with flagellin [20], underscoring the cell type-dependence of these effects. In microglia, the brain-resident macrophages, polyinosine-polycytidylic acid (poly(I∶C), a synthetic analog of dsRNA and a TLR3 ligand) and LPS seem to inhibit HIV-1 infection in an IFN regulatory factor 3-dependent manner [21]. In addition, poly(I∶C)-induced restriction of HIV-1 infection in macrophages has been shown, albeit without clear mechanistic insights [22], [23]. Interestingly, a recent study suggested a role for an unidentified, novel mechanism in TLR3-, TLR4- and TLR7-mediated reduction of HIV-1 infection in primary macrophages [24], and a TLR3 polymorphism has just been shown to have a higher frequency among HIV-1-exposed seronegative individuals, and to confer some degree of natural resistance to HIV-1 infection [25].

Macrophage stimulation through TLRs also modulates the expression of microRNAs (miRNAs) [26]–[28], temporally and spatially regulated non-coding RNA oligonucleotides that are potent regulators of gene expression. Mature miRNAs specifically bind to 3′ untranslated regions (3′UTR) of target mRNAs leading to either mRNA degradation or inhibition of translation. In addition to their roles in cellular development, differentiation and cancer, miRNAs are key for the development and functional regulation of the immune system and inflammatory responses [29]–[32]. Since inflammatory stimuli have a profound influence on gene expression, the innate immune response has the potential to regulate cellular miRNA levels, and it has been shown that stimulation through specific TLR ligands can modulate miRNA expression profiles [26]–[28]. Moreover, it was recently reported that some miRNAs (miR-28, miR-150, miR-223 and miR-382), which may directly target the 3′UTR of the viral mRNAs [33], are more abundant in monocytes than in macrophages and may contribute to the reduced susceptibility of monocytes to HIV-1 infection [34], although further mechanistic studies are probably needed [35]. In addition, miR-29a has been reported to inhibit viral replication [36], [37], and miR-198 and −27b seem to restrict HIV-1 replication in monocytes and CD4^+^ T cells, respectively, by repression of cyclin T1 expression, an important co-factor for HIV-1 Tat [38], [39]. Lastly, some studies have profiled miRNAs in various cell populations infected *in vitro* [40], [41] to obtain information about the effects of HIV-1 infection in miRNA expression, and in clinical samples to evaluate potential relationships with clinical parameters or status (such as CD4^+^ T cell counts and viral load, or among long-term non-progressors, naïve and multiple-exposed uninfected individuals) [42], [43].

Several studies have characterized the importance of miR-155 to establish appropriate immune responses (reviewed by O'Connell et al. [31]). miR-155 has been shown to increase in the human monocytic cell line THP1 and in murine bone marrow-derived macrophages in response to stimulation with LPS and poly(I∶C) [27], [28], respectively, and it seems to play a fundamental role in the macrophage inflammatory profile. In addition, in dendritic cells it seems that miR-155 targets the transcription factor PU.1, thus down-regulating the expression of dendritic cell-specific intercellular adhesion molecule-3 grabbing non-integrin (or DC-SIGN) [44], which is known to bind to the HIV-1 envelope glycoprotein gp120 and to participate in the process of trans-infection [45]. However, it is not known whether miRNA modulation, and specifically miR-155 levels, may play a role in the suppression of HIV-1 infection in TLR-activated macrophages.

In this study, we found that stimulation of normal human monocyte-derived macrophages (MDMs) from multiple donors with poly(I∶C) or LPS, but not with the ligands for TLR7 (Imiquimod) or TLR9 (CpG), consistently abrogates their susceptibility to infection by CCR5-using, macrophage-tropic HIV-1, and by vesicular stomatitis virus glycoprotein (VSV-G)-pseudotyped virions, suggesting a post-entry restriction mechanism. We also demonstrate that miR-155 is selectively up-regulated upon TLR3/4- but not TLR7-stimulation, and that specific inhibition of miR-155 partially restores HIV-1 infection in poly(I∶C)-stimulated MDMs. In addition, ectopic expression of miR-155 greatly reduces HIV-1 infection in MDMs and cell lines. Both TLR3 stimulation and ectopic miR-155 expression induce an accumulation of late reverse transcription (RT) products and an absence of or large reduction in integrated pro-viruses. Finally, increased miR-155 levels seem to reduce the mRNA and protein levels of several HIV-1 dependency factors (HDFs) that participate in trafficking and/or nuclear import of the pre-integration complex (PIC), suggesting a potential mechanism for its novel anti-HIV-1 effect.

## Materials and Methods

### Ethics statement

De-identified human monocytes from healthy blood donors were obtained from the University of Pennsylvania's Human Immunology Core (operating under the supervision of the University of Pennsylvania's Institutional Review Board). We did not have any interaction with human subjects or protected information, and therefore no informed consent was required. All studies were approved and supervised by Drexel University's Institutional Review Board.

### Cells and cell culture

MDMs were generated by culturing monocytes in Dulbecco's modified Eagle's Medium (DMEM) supplemented with 10% heat-inactivated fetal bovine serum (ΔFBS; BenchMark FBS, Gemini Bio-Products, West Sacramento, CA; certified for low endotoxin and hemoglobin levels), antibiotics, and 16 U/ml macrophage colony-stimulating factor (eBioscience, San Diego, CA) for 7 d. Human embryonic kidney 293T cells were cultured in DMEM supplemented with 10% ΔFBS and antibiotics. Human osteosarcoma cells stably expressing CD4 and CCR5 (HOS-CD4-CCR5; obtained through the AIDS Research and Reference Reagent Program, Division of AIDS, NIAID, NIH [ARRRP] from Dr. Nathaniel Landau) [46], and human glioblastoma U87 cells stably expressing CD4 and CCR5 (U87-CD4-CCR5; obtained through the ARRRP from Drs. HongKui Deng and Dan Littman) [47], were cultured as previously described [48].

### Production of viruses and pseudoviruses for infection

We produced a fully-infectious, firefly luciferase-reporter recombinant virus by transfecting 293T cells by calcium phosphate precipitation method (ProFection Mammalian Transfection System; Promega, Madison, WI) with the pNL-ADA-Fluc provirus, which we constructed by introducing the envelope glycoprotein of the CCR5-using, macrophage-tropic isolate HIV-1_ADA_ [49] into the pNL-HxB-Fluc provirus [50]. Work with fully-infectious virus was performed in a Bio-safety level 3 laboratory under standard operating procedures. Production of single-round infectious, luciferase-reporter pseudotypes was performed in 293T cells as previously described [48], [51], using the envelope-expression vectors of CCR5-using, macrophage-tropic BaL [52] or BR [48], [51] HIV-1 envelope glycoproteins, or a VSV-G expression vector. Supernatants containing viral stocks were collected 2 d post transfection, clarified, aliquoted, and stored at −80°C.

For infections, a 1∶2 dilution of viral stocks was added to target cells for 5–6 h. Cells were then washed twice with PBS and incubated with fresh media for 5 d (fully-infectious viruses) or 2–3 d (pseudoviruses), before quantitating infectivity by measuring luciferase activity in cell lysates (Luciferase assay system, Promega), as previously described [48], [51].

### TLR stimulation

Fully differentiated MDMs were incubated for 14–16 h in medium containing selected TLR ligands (all from InvivoGen, San Diego, CA; used at the manufacturer's recommended optimal concentrations), as follows: TLR2, heat-killed *Listeria monocytogenes* (10^8^ PFU/ml); TLR3, dsRNA analogue poly(I∶C) of a high molecular weight (average size of 1.5–8 kb) (10 µg/ml); TLR4, LPS (10 µg/ml); TLR7, Imiquimod (5 µg/ml); and TLR9, unmethylated CpG (10 µg/ml). After incubation, supernatants were collected, clarified and stored, and cells were washed and used for infection or additional tests.

### Immunofluorescence microscopy

MDMs were cultured in glass slides for 16 h in medium alone or with TLR ligands, and immediately after stimulation, stained with goat anti-human CD4 (AF-379-NA, R&D Systems, Minneapolis, MN) and mouse anti-human CCR5 (CTC8, R&D Systems) antibodies (Abs), followed by Alexa Fluor 488-conjugated anti-goat IgG and Alexa Fluor 594-conjugated anti-mouse IgG Abs (Invitrogen, Carlsbad, CA). DAPI (4–6′-diamidino-2-phenylindole) was used for nuclear staining. Images were obtained using an Olympus 1×81 spinning disk deconvolution fluorescent microscope and SlideBook 5.0 software (Intelligent Imaging Innovations, Denver, CO).

### Flow cytometry

For assessment of CD4 and CCR5 expression, MDMs were cultured in 6-well plates for 16 h in medium alone or with TLR ligands, and immediately after stimulation, gently collected from the plates using cold PBS, washed and stained with FITC-conjugated anti-CD4 and PE-conjugated anti-CCR5 Abs (eBioscience), or with the appropriate isotype control Abs. For analysis of TLR expression, unstimulated MDMs were collected as above and stained either under non-permeabilizing conditions (for surface staining) with PE-conjugated, anti-human TLR2 or TLR4 (both from eBioscience), or under permeabilizing conditions (for intracellular staining) with PE-conjugated, anti-human TLR3 or TLR9 (both from eBioscience), or PE-conjugated anti-human TLR7 (R&D Systems), or with the appropriate isotype control Abs. Immunofluorescence intensity was evaluated using a BD FACSCalibur flow cytometer (BD Biosciences, San Jose, CA) with CellQuest software (BD Biosciences), and data were analyzed using FlowJo flow cytometry analysis software (Tree Star, Ashland, OR).

### Enzyme-linked immunosorbent assays

Supernatants from unstimulated and TLR-stimulated MDMs were used for enzyme-linked immunoassays for quantitation of type I IFNs IFNα (PBL InterferonSource, Piscataway, NJ; and Thermo Scientific, Rockford, IL) and IFNβ (Thermo Scientific), pro-inflammatory cytokines tumor necrosis factor-α (TNF-α) and interleukin-6 (IL-6) (both from eBioscience), and β-chemokines macrophage inflammatory protein-1α (MIP-1α; SABiosciences, Frederick, MD), MIP-1β (Mabtech, Mariemont, OH) and regulated upon activation, normal T-cell expressed and secreted (RANTES; Peprotech, Rocky Hill, NJ), following manufacturer's instructions.

### MDM supernatant transfer experiment

Supernatants from untreated or TLR-stimulated MDMs were collected and added to fresh “naïve” macrophages for specified duration. After treatment with the conditioned supernatants, the MDMs were washed twice with PBS and infected with BAL pseudotypes for 48 h. Cells were then lysed and processed for luciferase activity.

### miRNA microarray

miRNA microarray analysis was performed in total RNA isolated using TRIzol (Invitrogen, Life Technologies Corp, Carlsbad, CA), following manufacturer's instructions, from macrophages from one donor cultured in the absence or in the presence of the specific ligands for TLR3, TLR4 and TLR7. miRNA expression profiles were evaluated at GenoSensor Corp. (Tempe, AZ) using GenoExplorer miRNA proprietary arrays with over 1300 miRNA probes (each in triplicate) for all described mature and most precursor human miRNA sequences (miRBase v13), in addition to multiple positive and negative control probes. Validated data were normalized according to the intensity of the signal of the positive control probes on each array chip. Signal intensity thresholds were defined as the array background plus 2 standard deviations, and at least two of the three repeating probes for each miRNA had to be above the signal intensity threshold for that miRNA to be further considered. Microarray data were deposited in the National Center for Biotechnology Information's Gene Expression Omnibus database (www.ncbi.nlm.nih.gov/geo) under accession number GSE34428.

### Quantitative RT-PCR

For quantitation of miR-155 levels, total RNA isolated from MDMs and cell lines (50 ng) was used for cDNA synthesis using TaqMan miRNA Reverse Transcription kit, followed by real-time PCR analysis using the miR-155-specific TaqMan miRNA Assay [53] (both from Applied Biosystems, Life Technologies Corp), performed in an Applied Biosystems 7300 Real Time PCR System. Results were calculated as relative quantitation compared to the levels of the U6 snRNA, using the comparative cycle threshold (Ct) method (also referred to as the 2^−ΔΔCt^ method) [54], [55], and were expressed as fold-change (mean ± SD) with respect to the appropriate biological control.

For relative quantitation of mRNA levels of IFNα1, IFNα2, IFNβ1 and several confirmed or potential HDFs, total RNA (500 ng) isolated from MDMs under different conditions was used for reverse transcription with Superscript III reverse transcriptase kit (Invitrogen), followed by real-time PCR using Taqman Gene Expression assays (Applied Biosystems), as per manufacturer's instructions. Results were calculated as relative quantitation compared to the levels of the 18S rRNA as endogenous control, using the comparative Ct method, and were expressed as fold-change with respect to the appropriate biological control.

### miR-155 inhibition

The Ambion Anti-miR miR-155 inhibitor that specifically binds to and inhibits human miR-155 (anti-miR-155), or the Cy3 dye-labeled Anti-miR scrambled negative control (anti-miR-scr, which does not target any known human miRNA) (Applied Biosystems), were transfected at an optimized concentration of 30 nM into MDMs using Lipofectamine RNAiMax transfection reagent (Invitrogen). At 24 h post-transfection, MDMs were washed with PBS and replaced with media alone or containing poly(I∶C) or Imiquimod for TLR3 and TLR7 stimulation, respectively, for 14–16 h. After stimulation, cells were used for infection or for RNA isolation, as described above. In order to estimate transfection efficiency of MDMs, images of cells transfected with Cy3 dye-labeled anti-miR-scr were obtained using an Olympus 1×81 spinning disk deconvolution fluorescent microscope and SlideBook 5.0 software.

### miR-155 over-expression in MDMs and cell lines

Over-expression of human miR-155 was achieved by transiently transfecting the non-viral plasmid miExpress Precursor microRNA pEZX-MR04 (Product ID: HmiR0358) containing the precursor sequence of hsa-miR-155 (pEZX-miR-155), or a scrambled sequence as negative control (pEZX-scrambled) (GeneCopoeia, Rockville, MD). Both plasmids encode for GFP expression that can be used for estimation of transfection efficiency. Plasmid DNA stocks were prepared using EndoFree plasmid maxi kit (Qiagen, Valencia, CA). HOS/CD4/CCR5 and U87/CD4/CCR5 cells were transfected with various amounts of pEZX-miR-155 or pEZX-scrambled plasmids (1–10 µg of DNA for 10^6^ cells per well in 6-well plates) using calcium precipitation, as indicated above. For MDMs, 6×10^5^ cells per well in 6-well plates were transfected with 6 µg of pEZX-miR-155 or pEZX-scrambled plasmids using jetPEI-Macrophage transfection reagent (PolyPlus-transfection, Illkirch, France), as per manufacturer's instructions. At 48 h post-transfection, cells were either infected or lysed for protein or RNA isolation. In addition, to estimate transfection efficiency of MDMs, images of cells untransfected or transfected with pEZX-miR-155 or pEZX-scrambled were obtained using an Olympus 1×81 spinning disk deconvolution fluorescent microscope and SlideBook 5.0 software.

### Quantitative PCR analysis of viral DNA products

Total DNA was isolated at 48 h post-infection from MDMs or cell lines using DNeasy Blood and Tissue kit (Qiagen). Detection of the early (RU5) and late (U5Ψ) RT products of HIV-1, and of 2-LTR circles (a short-lived, nuclear dead-end product), was performed by quantitative PCR as previously described [56], [57]. Primers and probes are listed in Table S1. As control for the amplification of 2-LTR circles, we treated MDMs with 5 µM or 20 µM Raltegravir (an HIV-1 integrase inhibitor; a gift from Dr. Simon Cocklin) for 24 h immediately after infection, and before extraction of DNA. For an absolute quantitation of early and late RT products, a serially diluted HIV-1 backbone plasmid pNL4.3 with known copy numbers, and no-template controls, were used in each PCR procedure to generate a standard curve, as previously described [58].

Integration of HIV-1 viral sequences was detected using a two-step Alu-LTR PCR as previously described [59], with minor modifications. Briefly, an initial non-kinetic amplification step was performed with 100 ng total DNA using Tth DNA polymerase (Promega) with previously described primers (Table S1) and the following conditions: 3 min hot-start at 94°C, followed by 35 cycles of denaturation at 94°C for 30 s, annealing at 50°C for 30 s, and extension at 72°C for 1 min and 40 s, and a single step final extension at 72°C for 3 min. The second-round, quantitative PCR was performed using 25 µl of the first amplification with the previously described conditions and primers, but with a modified probe (Table S1). For absolute quantitation of integrated pro-viral copy numbers, a known standard of serially diluted CEM cells infected with VSV-G pseudotyped, HIV-Δenv-hygromycin-GFP viruses (plasmid kindly provided by Dr. Robert Siliciano) was used, as previously described by Liszewski et al. [58].

To estimate the viral copy number per cell, we simultaneously performed detection of the housekeeping gene porphobilinogen deaminase (PBGD; accession number M95623) as endogenous control, in standards of known concentration of serially diluted PBMC DNA and in all samples, as previously described [56], allowing the estimation of the number of cells per well, and subsequently the calculation of copy number per cell. Relative quantitation of 2-LTR circles was determined by using CCR5 as endogenous control, as previously described [56]. PBGD and CCR5 primers and probes are listed in Table S1. All real-time PCR reactions were performed using TaqMan Universal Master Mix (Applied Biosystems), and were run in an Applied Biosystems 7300 Real Time PCR System.

### 3′ UTR target reporter assays

To validate potential miR-155 targets, 293T cells (which do not have detectable endogenous miR-155) were co-transfected in 6-well plates by calcium precipitation with: (i) 3 µg DNA of pEZX-miR-155 or the pEZX-scrambled control; and (ii) 3 µg DNA of miTarget microRNA 3′ UTR Target Sequence (pEZX-MT01) expression clones (GeneCopoeia), which contain the 3′ UTR sequence of interest (HmiT001349: LEDGF; HmiT000372: ADAM10; HmiT054432: TNPO3; HmiT023438: Nup153; and CmiT000001: a negative control 3′ UTR) inserted downstream of the firefly luciferase gene, and an independently-controlled Renilla luciferase gene to be used as normalization control, allowing functional validation of predicted miRNA targets. At 48 h post-co-transfection of 293T cells, cells were lysed and the regulatory effect of miR-155 on its potential target was assessed in cell lysates with a dual luciferase assay (Luc-Pair miR Luciferase assay; GeneCopoeia) as per manufacturer's instructions. Results were calculated as [(firefly/Renilla)_miR-155_/(firefly/Renilla)_scrambled_]×100, for each of the pEZX-MT01 clones, and are expressed as mean ± SD from four independent wells.

### Silencing of selected HDFs

MDMs were either untransfected or transfected for 48 h (using a 1∶1 ratio with LipoRNAiMax, Invitrogen) with Silencer Negative Control #2 siRNA (which has no significant sequence similarity to human gene sequences and is validated for use in human cells), or with Silencer Select siRNAs (two pre-validated siRNAs per target) for LEDGF, ADAM10 and Nup153 (all from Ambion), alone or in various combinations, following manufacturer's recommendations. Final concentration of each siRNA was 5 nM. Subsequently, cells were either lysed for western-blot analyses of protein levels, or infected and lysed for quantitation of infection by luciferase activity, or infected and lysed for DNA isolation and quantitation of viral RT products and integrated pro-virus.

### Western-blots

Whole cell lysates were prepared using RIPA buffer (Pierce Thermo Scientific) in MDMs at 48 h post TLR stimulation, post pEZX-miR-155 or -scrambled plasmid transfection, and post siRNA transfection. Protein amounts were estimated using a BCA assay (Pierce Thermo Scientific). Subsequently, 30–50 µg of total protein was loaded in each lane of a SDS-PAGE gel (BioRad) in denaturing conditions and then transferred to nitrocellulose membrane. Blots were probed with rabbit anti-human ADAM10 (1∶500 dilution) (Abcam, Cambridge, MA; ab84595), mouse anti-human LEDGF (1∶500 dilution) (Abcam; ab110023), mouse anti-human Nup153 (1∶300) (Abcam; ab24700), and mouse anti-human TNPO3 (1∶100 dilution) (Abcam; ab54353). All blots were also probed with rabbit anti-human GAPDH Ab (1∶5,000 dilution) (Cell Signaling, Danvers, MA) as loading control. All primary Abs were incubated overnight at 4°C as per manufacturer's instructions. Goat anti-mouse and goat anti-rabbit poly-HRP secondary Abs (Pierce Thermo Scientific) were used at 1∶5,000 dilution, as per manufacturer's instructions. Western blotting Chemiluminescence Luminol reagent (Santa Cruz Biotechnology; Santa Cruz, CA) was used as substrate and an Alpha Innotech FluorChem SP digital imaging system was used for capturing images and for the spot densitometry analyses. Chemoluminescent signals were then normalized with the loading control, and were expressed as percent with respect to unstimulated or untransfected controls.

### Statistical analyses

Statistical significance of experimental data was evaluated using SPSS Statistics software, with a *p* value equal to or less than 0.05 considered statistically significant.

## Results

### HIV-1 infection is suppressed in MDMs stimulated through TLR3 or TLR4, but not TLR7 or TLR9

**Figure 1:**
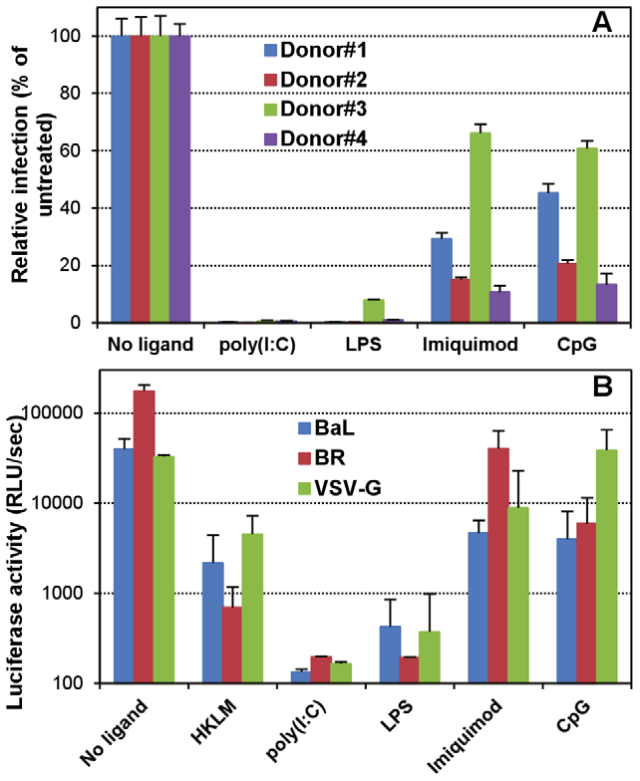
TLR3 and TLR4 stimulation abrogates susceptibility to HIV-1 infection of primary macrophages. (A) MDMs were untreated or incubated for 16 h with specific ligands for TLR3 (10 µg/ml poly(I∶C)), TLR4 (10 µg/ml LPS), TLR7 (5 µg/ml Imiquimod) or TLR9 (10 µg/ml CpG), and, after washing, infected with a fully-infectious, luciferase-reporter recombinant virus containing the macrophage-tropic ADA envelope glycoprotein. Inocula were removed after 4–6 h and cells were maintained for 5–6 days before quantitating infection through luciferase (luc) activity in cell lysates. Results with cells from four normal donors are shown as relative infection compared to untreated (mean ± standard deviation [SD]). (B) MDMs were untreated or incubated overnight with specific ligands for TLR2 (10^8^ PFU/ml heat-killed Listeria monocytogenes, HKLM), TLR3, TLR4, TLR7 or TLR9 (as indicated above), and then infected with luc-reporter, lentiviral particles pseudotyped with the macrophage-tropic BaL or BR HIV-1 envelope glycoproteins, or with VSV-G. Luc activity was measured 2–3 days post-infection and results from a representative experiment are shown as actual luc activity in cell lysates measured as relative light units per second (mean ± SD).

Macrophages are usually susceptible to HIV-1 infection *in vivo* and *in vitro* after their differentiation from blood monocytes [49], [60], [61], and the MDM model is widely used to address the regulation of HIV-1 infection in macrophages. Using this model, modulation of receptor expression, as well as increases in β-chemokines or IFNα production have been proposed, among others, as mechanisms that contribute to the reduced HIV-1 infection in LPS-stimulated MDMs [12]–[18], while no clear mechanism has been reported for poly(I∶C)-induced restriction of HIV-1 infection in MDMs [22]–[24]. We found that stimulation of MDMs from various donors with poly(I∶C) or LPS consistently suppressed HIV-1 infection, while Imiquimod and CpG reduced infectivity to varying extent depending on the donor, but never to levels similar to those achieved with poly(I∶C) and LPS (Figure 1A). In addition, we found abrogation – or almost complete suppression – of infection by pseudoviruses containing the BaL or BR envelope glycoproteins in MDMs stimulated through TLR3 or TLR4, with only partial reduction observed in cells stimulated through TLR2, TLR7 or TLR9 (Figure 1B). Remarkably, identical results were found when MDMs were infected with VSV-G-pseudotyped virions, suggesting that the differential effects observed did not involve alterations in the process of viral entry, but were rather due to post-entry events. This is in agreement with some previous studies that had also reported inhibition of infection of VSV-G-pseudotyped HIV-1 virions by TLR agonists (poly(I∶C) and LPS) in primary human myeloid cells [62], [63].

### Expression of TLRs and viral receptors and cytokine/chemokine production

Despite the fact that we observed the same modulation of infection by HIV-1 envelope- and VSV-G-pseudotyped virions, we wanted to investigate whether the TLR stimulation had any effect on CD4 and/or CCR5 expression in MDMs. Using immunofluorescence microscopy, we did not observe any difference in the expression of CD4 or CCR5 between untreated and poly(I∶C)-, LPS- or Imiquimod-treated MDMs (Figure S1A–D). We also utilized flow cytometry with staining performed under non-permeabilizing conditions, and found again an absence of differential CD4 or CCR5 expression among unstimulated and TLR ligand-stimulated MDMs (Figure S1E). In addition, flow cytometry analysis under non-permeabilizing (TLR2, TLR4) or permeabilizing (TLR3, TLR7, TLR9) conditions demonstrated that all TLRs investigated had detectable expression in primary human MDMs (Figure S2), albeit, as expected, with some donor-to-donor variability.

**Figure 2:**
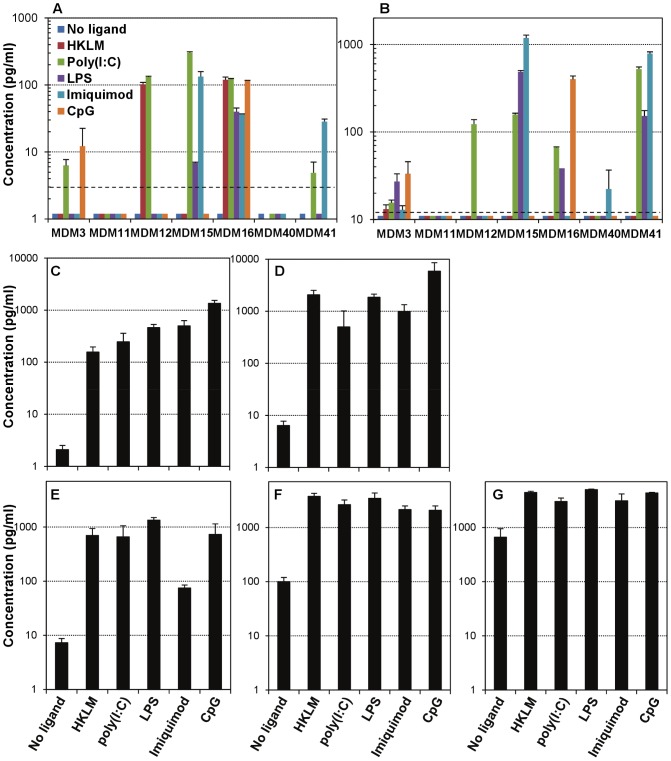
Production of type I IFNs, pro-inflammatory cytokines and β-chemokines after TLR stimulation in macrophages. MDMs were cultured in quadruplicate for 16 h unstimulated or with the ligands for TLR2, TLR3, TLR4, TLR7 or TLR9, and then supernatants were collected, clarified, aliquoted and stored at −80°C until use. Commercial enzyme-linked immunoassays were used for quantitation of type I IFNs IFNα (PBL InterferonSource and Thermo Scientific) (A) and IFNβ (Thermo Scientific) (B), pro-inflammatory cytokines TNF-α (C) and IL-6 (D) (both from eBioscience), and β-chemokines MIP-1α (CCL3) (E) (SABiosciences), MIP-1β (CCL4) (F) (Mabtech) and RANTES (CCL5) (G) (Peprotech). All samples were analyzed in duplicate. Multiple donors are shown for type I IFNs and one representative donor is shown for pro-inflammatory cytokines and β-chemokines. Results are shown as mean ± SD of duplicate cultures. Dashed lines indicate the limit of detection of the assays for quantitation of type I IFNs.

We next investigated the potential role of type I IFNs, pro-inflammatory cytokines and β-chemokines in the reported effects of TLR agonists in HIV-1 infection of MDMs. We first measured the production of type I IFNs and found that neither IFNα nor IFNβ were consistently produced to high levels, with robust increases in IFNα or IFNβ production dispersedly observed in a ligand- and donor-dependent manner (Figure 2A–B). Next, we measured MDM production of pro-inflammatory cytokines TNF-α and IL-6 and β-chemokines CCL3–5 (MIP-1α, MIP-1β and RANTES, respectively), and found that all ligands induced robust increases in the production of all of them, confirming that the MDMs were indeed responding to the TLR stimulation. However, despite small variations between ligands, their levels did not correlate with the observed differences in susceptibility to infection (Figure 2C–G). In addition, we quantitated the levels of type I IFN mRNAs induced by the ligands. In agreement with the lack of consistent robust production of IFNα or IFNβ into the culture supernatants, we found that IFNα1, IFNα2 and IFNβ mRNAs increased, although in a donor-dependent manner, in response to selected TLR stimulation (Figure S3).

Finally, we found that culture supernatants from MDMs stimulated with poly(I∶C), LPS or Imiquimod all induced a similar reduction in susceptibility to HIV-1 infection in fresh “naive” macrophages (Figure S4), ranging between 60 and 88% of infection observed in the same cells exposed to supernatants from unstimulated MDMs.

### Differential miRNA expression in TLR-stimulated MDMs

To gain an insight into the potential differences in miRNA expression profiles between unstimulated and poly(I∶C)-, LPS- and Imiquimod-stimulated MDMs, we performed miRNA microarray analyses using GenoExplorer miRNA Expression Analysis (GenoSensor). Validated data (Gene Expression Omnibus database accession number GSE34428) showed that TLR3-, 4- and 7-stimulated MDMs displayed overlapping but distinct changes in miRNA expression profiles compared to unstimulated cells (Figure S5A–B). We identified a subset of four miRNAs that were significantly up-regulated in cells stimulated through TLR3 and TLR4 but not through TLR7 and could potentially play a role in modulating susceptibility to HIV-1 infection in this system. Among them, we focused on miR-155 since it displayed the highest up-regulation in confirmatory quantitative PCR (not shown), and because of its roles in macrophage inflammatory and immune responses.

**Figure 3:**
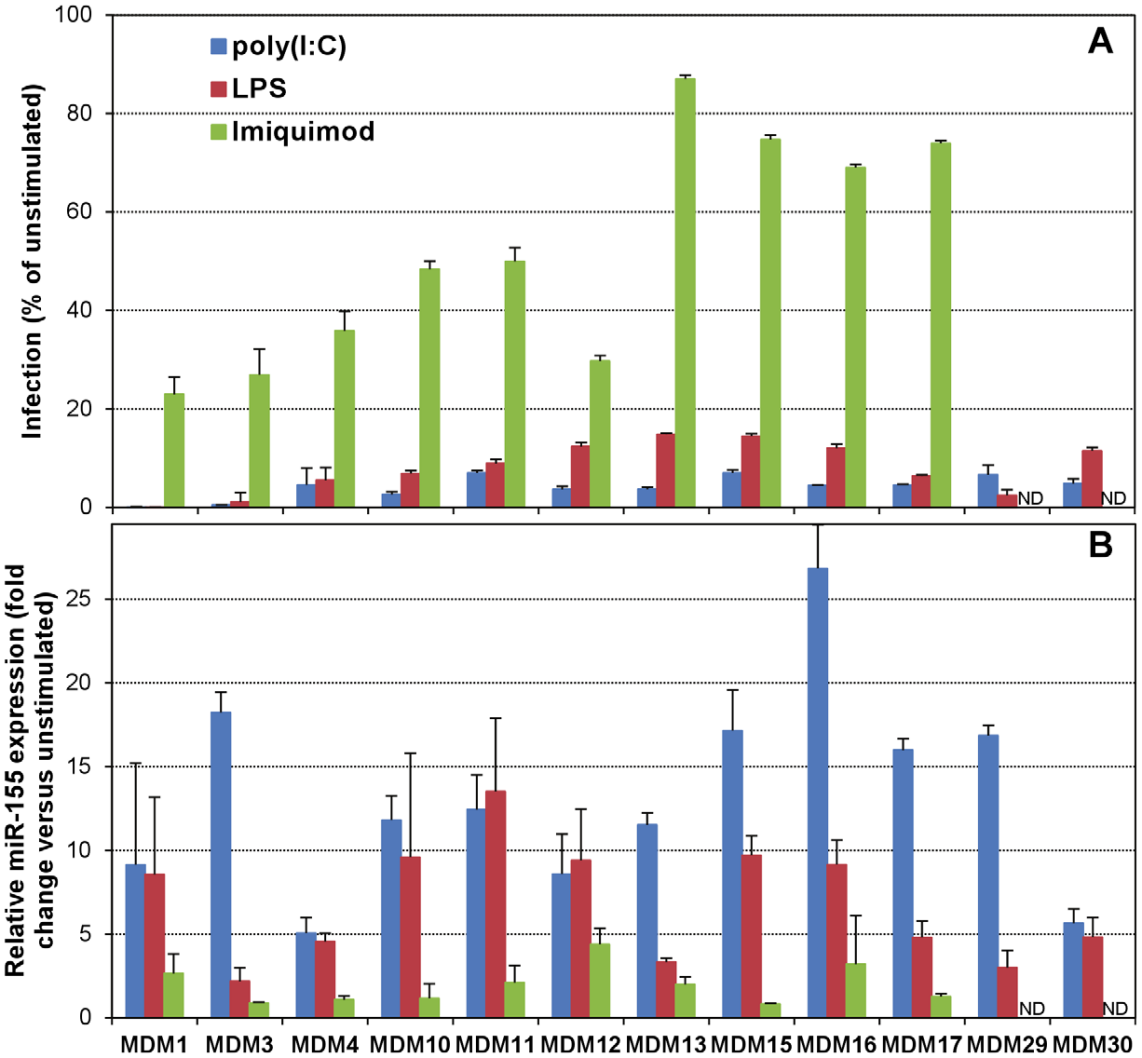
Stimulation through TLR3 and TLR4, but not TLR7, increases miR-155 levels in primary macrophages. MDMs from 12 different donors were cultured for 16 h unstimulated or with the ligands for TLR3, TLR4 or TLR7, and then used for infection with BaL pseudotypes (A) or for RNA isolation with Trizol (Invitrogen) for miR-155 quantitation (B). Infection was evaluated in 8 replicates as percent luc activity compared to unstimulated MDMs (mean ± SD). Relative miR-155 levels were evaluated in duplicate in total RNA using TaqMan miRNA Reverse Transcription kit and Universal PCR Master Mix, and the miR-155-specific TaqMan miRNA Assay (Applied Biosystems), and results were calculated relative to the U6 snRNA levels using the comparative Ct method (also referred to as the 2^−ΔΔCt^ method), and expressed as fold-change (mean ± SD) with respect to the unstimulated control. ND, not determined.

We then determined the relative infection and miR-155 levels upon TLR stimulation in MDMs from a large cohort of normal donors. Despite the expected variability in their response to stimulation among primary cells from multiple donors, poly(I∶C)- and LPS stimulation in MDMs consistently resulted in abrogation or almost complete suppression of infection, which was never observed with Imiquimod stimulation (Figure 3A; Table S2). In addition, poly(I∶C) and LPS stimulation of MDMs generally resulted in robust increases in the levels of miR-155, mostly absent in Imiquimod-stimulated cells, as compared to unstimulated MDMs (Figure 3B; Table S2). Although a similar miR-155 increase in response to poly(I∶C) and LPS was observed in cells from some donors, a more robust induction with poly(I∶C) than with LPS was found in most of them, resulting in a statistically significant difference in miR-155 levels, as determined by paired Student's t test (Table S3). Taking together all infection and miR-155 data from poly(I∶C)-, LPS- and Imiquimod-stimulated MDMs, we performed curve estimation using a logarithmic regression model with miR-155 levels as independent variable and relative infection data as dependent variable, and found that the model is a good fit for the data (R^2^ = 0.48, p<0.001) (Figure S6A), which suggests that susceptibility to infection is determined, at least in part, by miR-155 levels.

These results suggested that poly(I∶C)- and LPS-induced suppression of HIV-1 infection in MDMs is related with their ability to induce miR-155.

### miR-155 contributes to the anti-HIV-1 effects of poly(I∶C)

**Figure 4:**
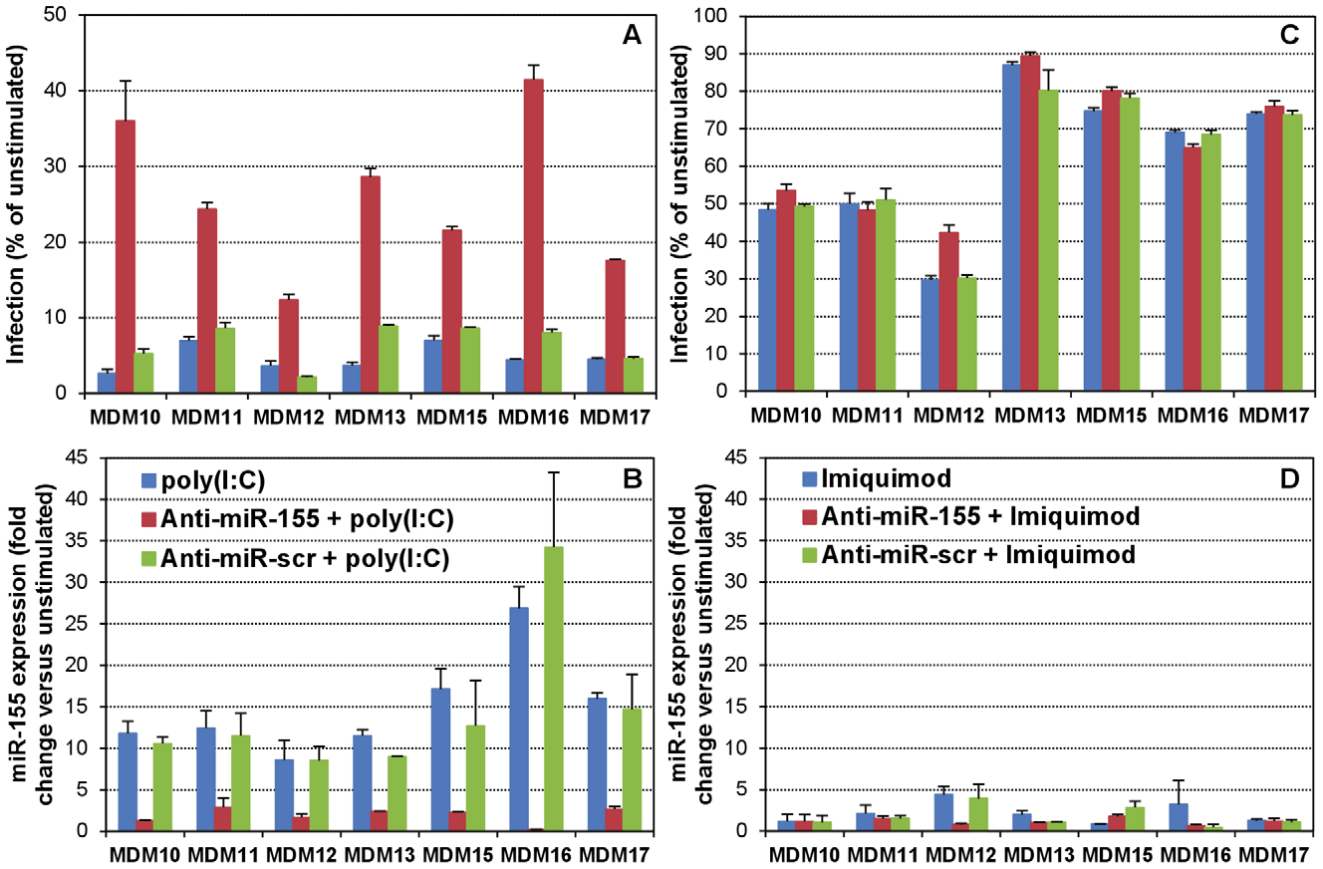
Inhibition of miR-155 in TLR3-stimulated macrophages increases their susceptibility to HIV-1 infection. MDMs from seven different donors were transfected for 24 h (LipoRNAiMax, Invitrogen) with 30 nM anti-miR-155 antagomir or a scrambled control that doesn't target any known human miRNA (anti-miR-scr) (Applied Biosystems), and then stimulated with poly(I∶C) (A and B) or Imiquimod (C and D) for 16 h, before either infecting with BaL pseudotypes (A, C) or isolating RNA for miR-155 quantitation (B, D). Infection was evaluated in 8 replicates as percent luc activity compared to untreated, unstimulated MDMs (mean ± SD). Relative miR-155 levels were evaluated as in Figure 3 and results are shown as fold-change (mean ± SD) with respect to the untreated, unstimulated MDMs.

To experimentally define whether the poly(I∶C)-induced increase in miR-155 levels contributed to the observed anti-HIV-1 effects upon TLR3 stimulation, we transfected MDMs with a specific anti-miR-155 inhibitor or a scrambled negative control (anti-miR-scr, which does not target any known human miRNA) before TLR stimulation. In general, we observed a very good efficiency of transfection of MDMs with the Ambion Cy3 dye-labeled anti-miR-scr (used as suggested by the manufacturer at an optimized concentration of 30 nM with Lipofectamine RNAiMax transfection reagent) (Figure S7). Remarkably, anti-miR-155 pre-treatment resulted in a statistically significant increase in relative infection of poly(I∶C)-stimulated MDMs from multiple donors (poly(I∶C), 4.7±1.7%; anti-miR-155+poly(I∶C), 26.0±10.2%; paired Student's t test, *p*<0.01), while anti-miR-scr pre-treatment had no effect (6.6±2.6%) (Figure 4A). The augmented susceptibility to infection induced by anti-miR-155 was concurrent with an abolishment of the increase in miR-155 levels observed in poly(I∶C)-stimulated MDMs (Figure 4B; poly(I∶C), 15.3±6.9 fold change; anti-miR-155+poly(I∶C), 1.9±1.0 fold change; paired Student's t test, *p*<0.01), while anti-miR-scr pre-treatment again had no effect (13.9±9.1 fold change). In addition, the anti-miR-155 pre-treatment in Imiquimod-stimulated MDMs failed to induce any changes (Figure 4C–D), except in MDM12 cells, in which a small increase in miR-155 level had been observed (Figure 3B), and anti-miR-155 pre-treatment resulted in a slightly increased infection concurrently with an abolishment of the small miR-155 induction.

Finally, treatment of MDMs with anti-miR-155 did not result in an alteration in the levels of type I IFN mRNAs induced by TLR3 stimulation (Figure S3), suggesting that type I IFN induction and miR-155 increase are independent effects of poly(I∶C) in MDMs.

### Ectopic expression of miR-155 reduces HIV-1 infection in MDMs and cell lines

**Figure 5:**
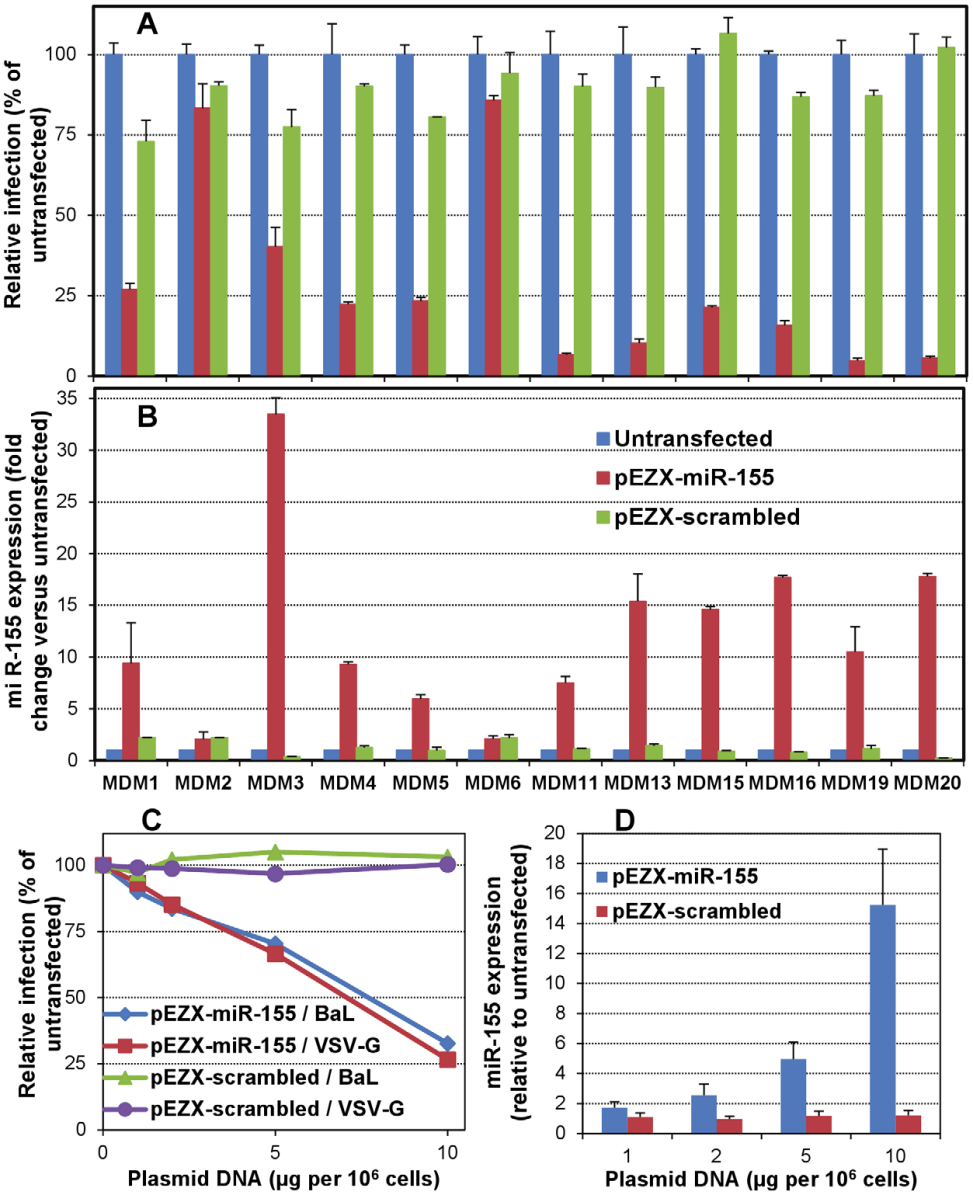
miR-155 over-expression in primary macrophages and cell lines decreases their susceptibility to HIV-1 infection. MDMs from 12 different donors were transfected with miR-155 expression plasmid (pEZX-miR-155) or scrambled control (pEZX-scrambled) (GeneCopoeia) using jetPEI-Macrophage transfection reagent (Polyplus), following manufacturer's instructions. After 48 h., cells were either infected with BaL pseudotype (A) to evaluate infection through luciferase activity as indicated above; or total RNA was isolated for miR-155 quantitation by real-time PCR (B) as indicated above. HOS-CD4-CCR5 cells were also transfected with various amounts of the miR-155 or scrambled plasmids using calcium precipitation, and then used for infection with BaL or VSV-G pseudotypes (C) or RNA isolation for miR-155 quantitation (D). Infection was evaluated in 8 replicates as percent luc activity compared to untransfected cells (mean ± SD). Relative miR-155 levels were evaluated as in Figure 3 and results are shown as fold-change (mean ± SD) with respect to the untransfected control.

To confirm that an increase in the levels of miR-155 in primary macrophages can have an effect on their susceptibility to HIV-1 infection, we performed over-expression experiments by transfecting MDMs with a miR-155-expressing plasmid (pEZX-miR-155) or a scrambled control (pEZX-scrambled). MDMs transfected with pEZX-miR-155 showed a remarkably lower HIV-1 infection than that observed in untransfected or pEZX-scrambled-transfected cells in 10 out of the 12 donors investigated (Figure 5A), with between 60 and 96% inhibition of infection. In addition, the reduced infectivity correlated with an increase in miR-155 levels in pEZX-miR-155-transfected cells from the same 10 donors, which was absent in untransfected or pEZX-scrambled-transfected cells (Figure 5B). MDM2 and MDM6 cells failed to show a decrease in HIV-1 infection but also lacked an increase in miR-155 levels upon transfection with pEZX-miR-155, suggesting that the absence of an effect on susceptibility to infection was due to inefficiency of transfection of MDMs from these two donors.

Since primary cells in general, and macrophages in particular, are known to be partially refractory to transfection, we evaluated GFP expression by fluorescent microscopy in MDMs transfected with pEZX-miR-155 and pEZX-scrambled control (Figure S8), confirming that the large biological effects observed in cells from a majority of donors are probably related with a generally high efficiency of transfection. Curve estimation using a logarithmic regression model of miR-155 levels (independent variable) and relative infection data (dependent variable) indicated a good fit of the model (R^2^ = 0.514, p<0.01) (Figure S6B), despite the presence of an outlier that resulted in a moderate multiple correlation coefficient (R = 0.717). Removal of the outlier increased even further the goodness of the model (R^2^ = 0.804, p<0.001), suggesting that susceptibility to infection in transfected MDMs is determined, in large part, by the miR-155 levels achieved upon transfection.

Furthermore, transfection of HOS-CD4-CCR5 cells with increasing amounts of pEZX-miR-155 plasmid, but not with pEZX-scrambled control plasmid, resulted in a dose-dependent decrease in infection with both HIV-1 envelope- and VSV-G-pseudotyped viruses, which correlated with a dose-dependent increase in miR-155 levels (Figure 5C–D). Similar effects were observed in U87-CD4-CCR5 cells (data not shown). These results suggest that miR-155-induced anti-HIV-1 effects are cell type-independent, observed in immune and non-immune system cell types, and are mediated by alteration of one or more post-entry steps in the viral life cycle.

### miR-155 induction leads to an accumulation of late viral transcripts and reduced integration

**Figure 6:**
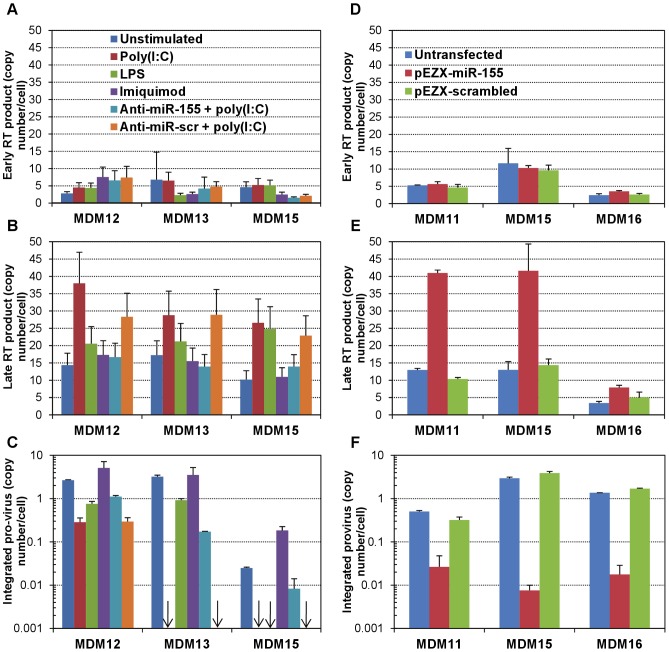
Increase in miR-155 leads to late RT products accumulation and greatly reduced integration. Total DNA was isolated at 48 h post-infection from: (i) BaL pseudotype-infected MDMs (from three different donors) that were unstimulated, or stimulated with poly(I∶C), LPS or Imiquimod, and in the case of poly(I∶C), untransfected or transfected (prior to stimulation) with anti-miR-155 or anti-miR-scr (A–C); (ii) BaL pseudotype-infected MDMs (from three different donors) that were untransfected or transfected (prior to infection) with miR-155 expression plasmid (pEZX-miR-155) or scrambled control (pEZX-scrambled) (D–F). Real-time PCR was performed in total DNA for the absolute quantitation of early RT product (RU5) (A,D), late RT product (U5Ψ) (B,E) and integrated pro-virus (C,F), as described in Materials and Methods. Copy numbers per cell were calculated using PBGD as endogenous control to normalize for DNA recovery and number of cells, and are shown as mean ± SD from two independent quantitations. Arrows indicate lack of amplification in real-time PCR, implying levels below the limit of detection of the technique.

To define the potential mechanism(s) involved in anti-HIV-1 effects induced by higher levels of miR-155 both in the context of TLR3 engagement and during over-expression experiments, we performed absolute quantitation of early and late RT products (RU5 and U5Ψ, respectively), and integrated pro-viral DNA by real-time PCR. No differences in the copy number per cell of early RT products were observed between unstimulated and poly(I∶C)-, LPS- or Imiquimod-stimulated MDMs of multiple donors (Figure 6A). However, poly(I∶C)-stimulated MDMs consistently displayed an accumulation of late RT products compared to unstimulated or Imiquimod-stimulated cells (Figure 6B), while LPS-stimulated cells did not consistently show this increase in the copy number per cell of late RT product. Moreover, integrated pro-viral genomes were readily detected in unstimulated and Imiquimod-stimulated MDMs but were undetectable in 2 out of 3 poly(I∶C)-stimulated MDMs (with a 10-fold decrease in copy number per cell in the remaining donor), and in 1 out of 3 LPS-stimulated MDMs (with reduced copy numbers per cell in the other two) (Figure 6C). The greater consistency observed in these effects at the level of viral DNA detection with poly(I∶C), as compared to with LPS, could relate with the more consistent and robust effect of poly(I∶C) in increasing miR-155 levels across the large number of donors investigated. Finally, while anti-miR-scr pre-treatment of poly(I∶C)-stimulated MDMs did not alter detection of early RT, late RT or integrated viral DNA forms with respect to poly(I∶C) alone, the specific anti-miR-155 antagomir completely reverted the poly(I∶C)-induced accumulation of late RT products in all three donors investigated, and also remarkably increased the amounts of integrated pro-viruses in all three donors (to similar copy numbers to those found in unstimulated MDMs; Figure 6B–C). Therefore, inhibition of the poly(I∶C)-induced miR-155 increase restored the capacity of the virus to complete nuclear import and integration, and prevented the accumulation of late RT product.

Similarly, in the context of ectopic expression of miR-155, no change was observed in detection of early RT products; however, we found that miR-155 induced substantial increases in the copy number per cell of late RT products and large decreases (up to over 2 orders of magnitude) in the copy number per cell of integrated pro-virus (Figure 6D–F). None of these effects were observed in MDMs transfected with the pEZX-scrambled control plasmid, which were indistinguishable from the untransfected control. Lastly, an accumulation of late RT product and absence of detection of integrated pro-virus, with no change in early RT product, were found as well in pEZX-miR-155-transfected U87-CD4-CCR5 cells, while no change in detection of any of the viral products was observed in pEZX-scrambled control-transfected cells (data not shown). Thus, ectopic expression of miR-155 in MDMs and cell lines led to an accumulation of late RT product, in the presence of greatly decreased amounts of integrated pro-virus, resulting in an identical phenotype to that found in poly(I∶C)-stimulated MDMs.

To determine whether the large reduction in, or absence of integration and accumulation of late RT products indicated an absence of nuclear import of viral DNA, we performed real-time PCR for detection of 2-LTR circles, a short-lived, dead-end product of viral DNA that forms in the nucleus and serves as a marker of recent infection, completion of reverse transcription and successful nuclear import of viral DNA. 2-LTR circles were either undetectable or detected at very low levels in most of our MDM samples, regardless of stimulation or transfection. However, we treated MDMs with the integrase inhibitor Raltegravir and demonstrated the formation of 2-LTR circles under those conditions (Figure S9). Hence, it seems that higher levels of miR-155 induced by poly(I∶C) stimulation or ectopic expression may prevent nuclear import of viral DNA, resulting in an accumulation of late RT products in the cytoplasm of infected cells.

### miR-155 may target mRNAs of HDFs involved in trafficking/nuclear import of the pre-integration complex

To identify potential miR-155 targets that could be mediating the effects reported above, we used: miRGen (www.diana.pcbi.upenn.edu/miRGen) [64], an interface that provides access to unions and intersections of widely used target prediction programs (e.g., TargetScan [65]–[68] and MicroCosm Targets [69]–[72]), and to experimentally-supported targets; and miRWalk (www.umm.uni-heidelberg.de/apps/zmf/mirwalk/) [73], which features a newly developed algorithm that evaluates potential targets on the complete sequence of all known human genes, and also provides information from 8 established, 3′ UTR-based, miRNA prediction programs and validated targets. These *in silico* analyses suggested that several confirmed or potential HDFs [74], [75] (due to the cellular processes that they are involved in), may be targets for miR-155. Specifically, we identified importins α3 and α5 (KPNA4 and 1, respectively), various nucleoporins from the nuclear pore complex (Nup107, 153 and 358 [or RanBP2]), and the cellular transcriptional co-activator lens epithelium-derived growth factor (LEDGF)/p75 (or PSIP1), all of which may participate in trafficking and nuclear import of the viral pre-integration complexes (PICs).

**Figure 7:**
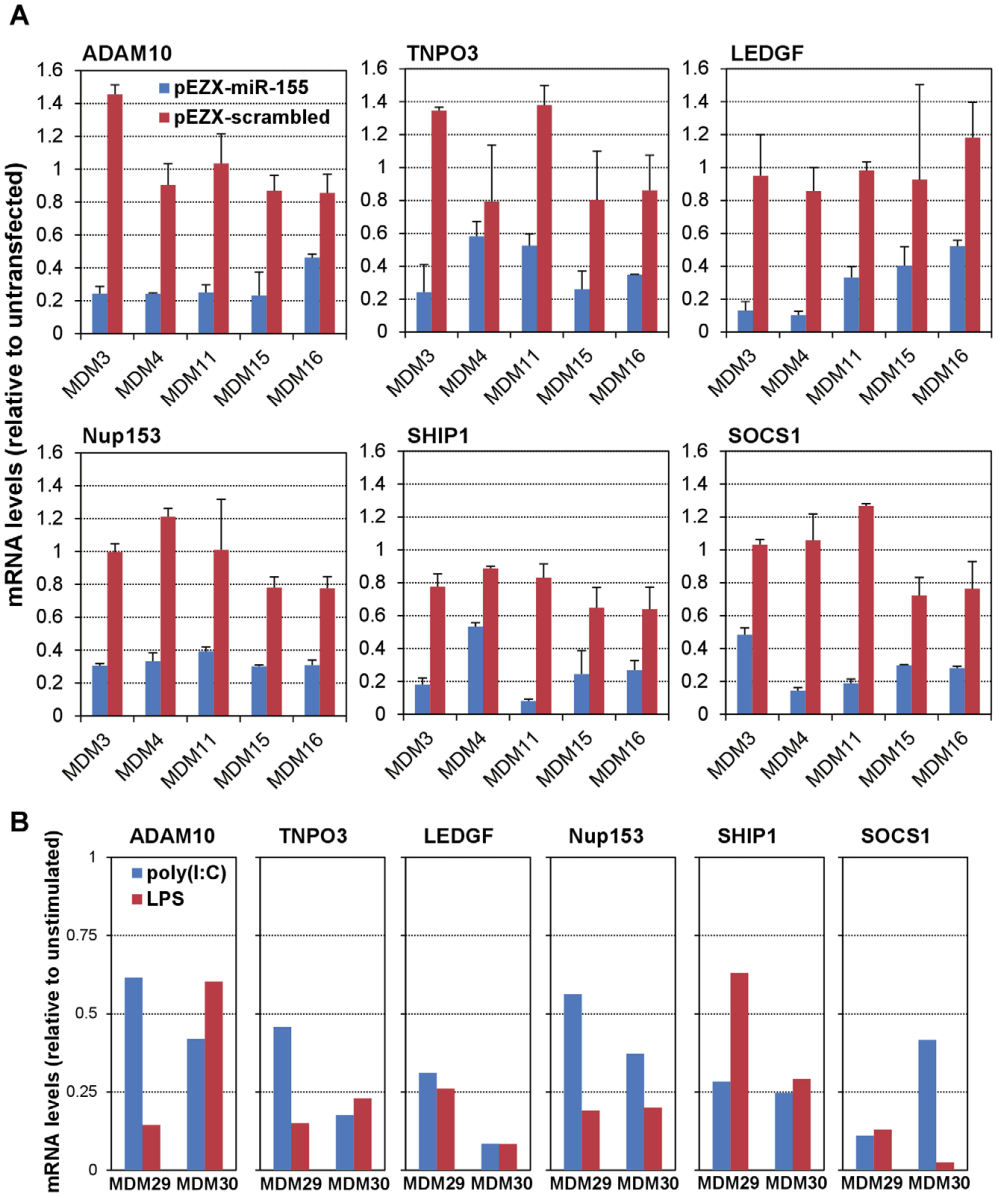
Increased miR-155 levels result in reduced mRNA levels of several HIV-1 dependency factors. We isolated total RNA at: (i) 48 h post-transfection of MDMs from five different donors with either pEZX-miR-155 or pEZX-scrambled control (A); and (ii) 24 h post-stimulation of MDMs from two different donors with poly(I∶C) or LPS (B); and then performed relative quantitation of mRNAs by real-time RT-PCR of two experimentally-demonstrated miR-155 targets (SHIP1 and SOCS1), and four not previously described, potential miR-155 targets (ADAM10, TNPO3, LEDGF and Nup153), using TaqMan Gene Expression assays (Applied Biosystems). Results were calculated using 18S rRNA as internal control and the comparative Ct method (also known as the 2^−ΔΔCt^ method), and are shown as fold-change (mean ± SD from two independent quantitations) with respect to the untransfected control in (A), or as fold-change (calculated from a single experiment) with respect to unstimulated in (B).

Therefore, we used total RNA from five donor MDMs over-expressing miR-155 to quantitate mRNAs for the identified potential targets, and for some additional HDFs such as importins α1 and β1 (KPNA2 and KPNB1, respectively), transportin-3 (TNPO3) and ADAM10 (a member of a disintegrin and metalloprotease family of proteins), which also seem to participate in the trafficking and/or nuclear import of PICs, although they were not predicted at all (KPNA2, KPNB1, TNPO3), or only by one algorithm (ADAM10), to be potential targets for miR-155. As expected, we found reduced mRNA levels of two validated miR-155 targets, inositol polyphosphate-5-phosphatase/145 kDa (INPP5D or SHIP1) [76] and suppressor of cytokine signaling 1 (SOCS1) [77], [78], which were evaluated as controls (Figure 7A). More importantly, ADAM10, TNPO3, NUP153 and LEDGF/p75 (PSIP1) all showed lower mRNA levels in miR-155-transfected than in untransfected MDMs, with a reduction larger than 2-fold in 3–5 of the MDMs investigated, while no changes were observed in total RNA from MDMs transfected with the pEZX-scrambled control (Figure 7A). Other mRNAs tested (KPNA1, KPNA2, KPNA4, KPNB1, Nup107, RanBP2) were either unchanged or not consistently reduced by at least 2-fold in at least three of the MDMs examined (data not shown).

Subsequently, we measured the mRNA levels of ADAM10, TNPO3, LEDGF and Nup153, and SHIP1 and SOCS1 as positive controls, in total RNA isolated from unstimulated, poly(I∶C)- and LPS-treated MDMs from two donors. As shown in Figure 7B, mRNA levels of LEDGF and TNPO3 were reduced greater than 2-fold by both poly(I∶C) and LPS, compared to unstimulated control, in the two donor MDMs, while the reduction was slightly less than 2-fold in one donor with poly(I∶C) for Nup153, and for one donor with poly(I∶C) and for the other donor with LPS, for ADAM10. Overall, the magnitude of the changes in mRNA levels of HDFs observed upon increased expression of miR-155, as well as upon TLR3 or 4 stimulation, are comparable to or larger than those reported in the literature for validated miRNA targets, e.g., such as in the studies describing SHIP1 and SOCS1 as miR-155 targets [76], [77], [79]. Thus, it seems possible that increased levels of miR-155 induced by either ectopic expression or TLR3 or TLR4 stimulation can reduce cellular susceptibility to HIV-1 infection by decreasing the expression levels of one or more HDFs, leading to the generation of an intracellular environment that is less conducive to productive HIV-1 infection.

### The 3′UTRs of LEDGF, ADAM10, TNPO3 and Nup153 are targeted by miR-155

**Figure 8:**
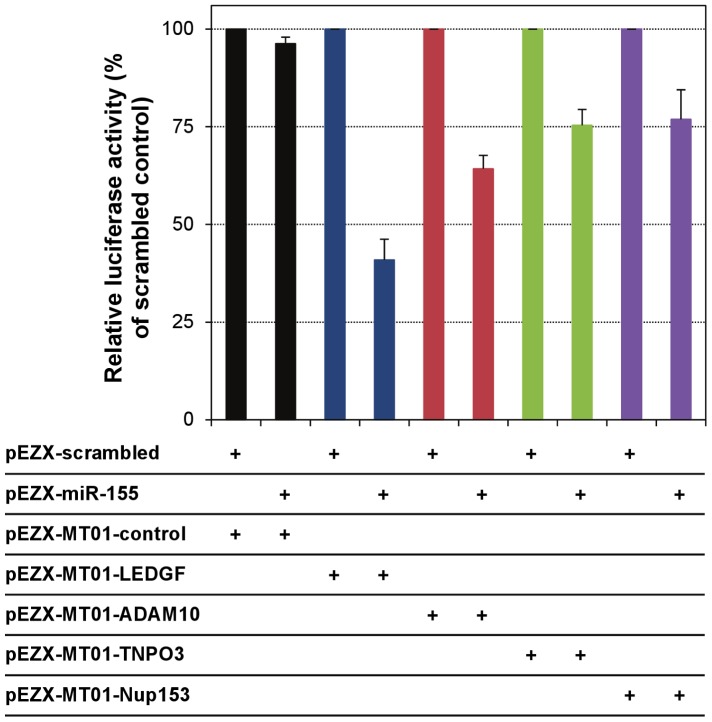
The 3′UTRs of LEDGF, ADAM10, TNPO3 and Nup153 are targeted by miR-155. 293T cells were co-transfected by calcium precipitation with: (i) pEZX-miR-155 or the pEZX-scrambled control; and (ii) with miTarget microRNA 3′UTR Target Sequence (pEZX-MT01) expression clones (GeneCopoeia), which contain the 3′UTR sequence of interest (LEDGF, ADAM10, TNPO3, Nup153 and a negative control 3′UTR) inserted downstream of the firefly luciferase, and an independently-controlled Renilla luciferase gene to be used as normalization control, allowing functional validation of predicted miRNA targets. At 48 h post-co-transfection of 293T cells, cells were lysed and the regulatory effect of miR-155 on its potential target was assessed in cell lysates with a dual luciferase assay. Results were calculated as [(firefly/Renilla)_miR-155_/(firefly/Renilla)_scrambled_]×100, for each of the pEZX-MT01 clones, and are expressed as mean ± SD from four independent wells.

To functionally validate that the 3′UTRs of candidate HDFs identified above contain a miR-155 target site that could mediate the miR-155-induced effects, we utilized a dual luciferase reporter expression system in 293T cells, which do not contain any detectable endogenous miR-155. In this system, the presence of the 3′UTR of interest downstream of the firefly luciferase will result in a modulation of expression only if that 3′UTR contains a miR-155 target site, while the independently-controlled Renilla luciferase expression will be unaffected, allowing the validation of the target and the quantitation of the inhibitory effect. Specificity was assessed by co-transfecting the pEZX-miR-155 with a pEZX-MT01 control reporter vector (which does not contain any known miRNA target site) and, as expected, no suppressive effect on firefly luciferase expression was observed, when compared to the co-transfection of pEZX-scrambled and pEZX-MT01 control (Figure 8). Firefly luciferase expression was remarkably reduced by miR-155, relative to that observed with the scrambled control, in the presence of the 3′UTR of LEDGF, and to a lower extent, with the 3′UTRs of ADAM10, TNPO3 and Nup153. To a large extent, these data seem to agree with the relative strength of the *in silico* prediction studies reported above, which strongly suggested LEDGF as a miR-155 target, and had a less robust prediction for ADAM10 and Nup153 as miR-155 targets.

### miR-155 increase in MDMs leads to reduced protein levels of selected HDFs

**Figure 9:**
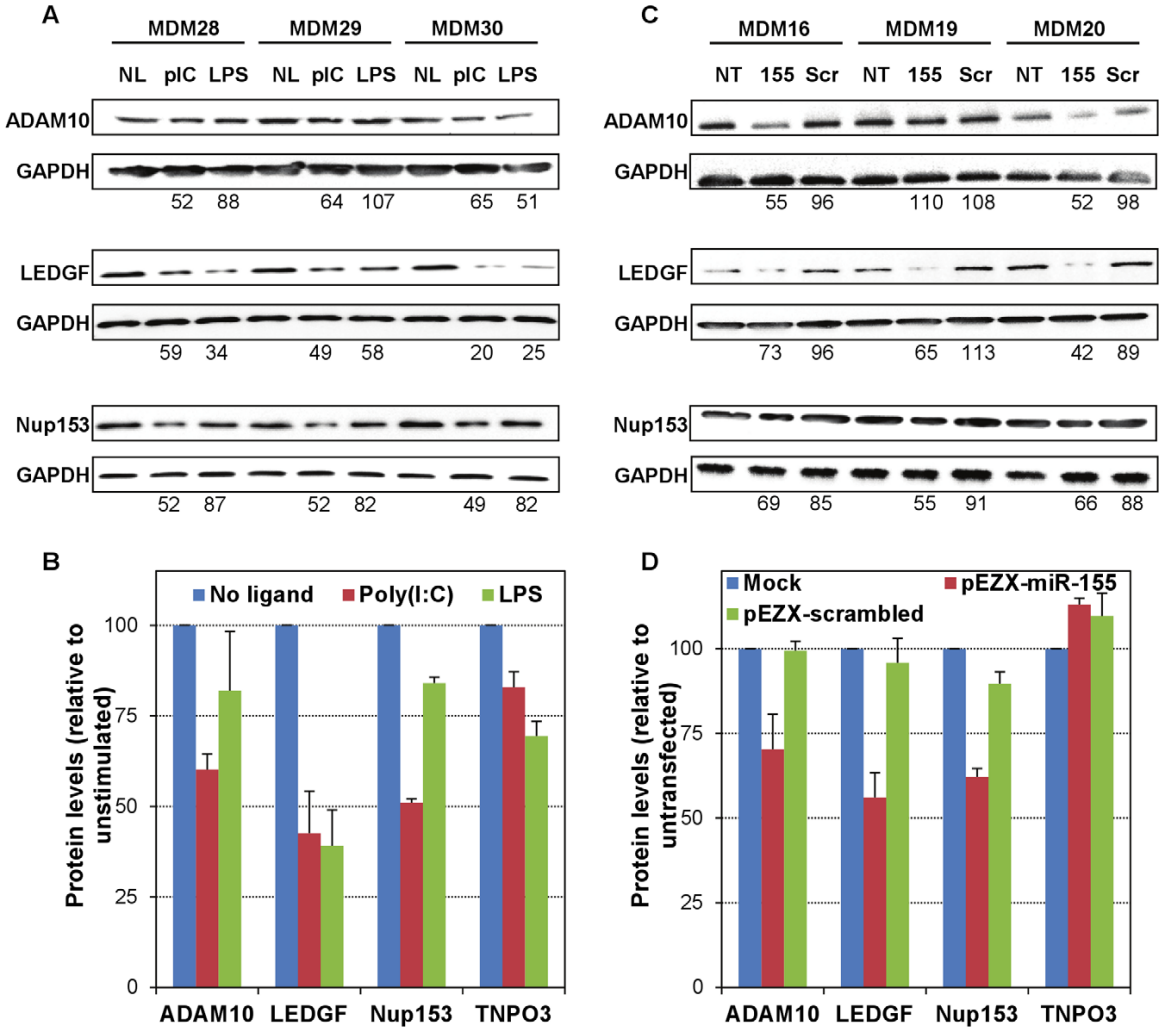
Increased miR-155 levels result in reduced protein levels of several HIV-1 dependency factors. We generated cellular lysates at: (i) 48 h post-stimulation of MDMs from three different donors with poly(I∶C) or LPS (A,B); and (ii) 48 h post-transfection of MDMs from five different donors with either pEZX-miR-155 or pEZX-scrambled control (C,D); and then performed western-blots using antibodies for ADAM10, LEDGF, Nup153 and TNPO3 (Abcam), and GAPDH (Cell Signaling) as loading control. Intensity of the chemoluminiscent signals was quantitated by densitometry analysis and normalized with the loading control, and is expressed as percent with respect to unstimulated (A,B) or untransfected (C,D) controls. (A) Poly(I∶C) and LPS decreased the levels of ADAM10, LEDGF, Nup153 and TNPO3 (not shown) to a different extent and with some donor-to-donor variation. (B) Effects of poly(I∶C) and LPS on expression of HDFs in MDMs from three donors, expressed as mean ± SD. (C) Ectopic expression of miR-155 led to decreased levels of ADAM10, LEDGF and Nup153, but not TNPO3 (not shown), with some donor-to-donor variation; three out of a total five donors are shown. (D) Effects of ectopic expression of miR-155 on expression of HDFs in MDMs from five donors, expressed as mean ± SD.

To confirm the functional validation of LEDGF, ADAM10, Nup153 and TNPO3 as targets of miR-155, we investigated their levels in MDMs both in the context of TLR stimulation and ectopic expression of miR-155. Western-blot analyses of cell lysates from unstimulated, poly(I∶C)- and LPS-treated MDMs from 3 donors demonstrated that both ligands resulted in robust decreases in the levels of LEDGF, and that poly(I∶C) reduced the levels of ADAM10 and Nup153 to a larger extent than LPS (Figure 9A–B), while both ligands had a less robust but detectable effect on the levels of TNPO3. Similarly, western-blot analyses of cell lysates from mock transfected, pEZX-miR-155- and pEZX-scrambled-transfected MDMs from 5 donors demonstrated that ectopic expression of miR-155 induced remarkable decreases in the levels of LEDGF, Nup153 and ADAM10, while the levels of TNPO3 were unaffected (Figure 9C–D). In addition, transfection of the scrambled control did not alter the level of expression of any of the HDFs investigated. These results demonstrate that increased levels of miR-155 not only lead to reduced mRNA levels of several HDFs, but also, and more relevantly, to reduced protein levels, confirming, at least for LEDGF, Nup153 and ADAM10, that they are bona-fide targets of miR-155 in primary macrophages. Regarding TNPO3, although TLR3/4 stimulation led to reductions both at the mRNA and protein levels and the 3′UTR reporter assay showed an effect of miR-155 on TNPO3's 3′UTR, ectopic miR-155 expression in macrophages only reduced its mRNA but not the protein levels, which could suggest that TNPO3 is also a miR-155 target but with a lower efficiency (perhaps due to imperfect complementarity) than the other HDFs. It is thus possible that this could relate with the fact that *in silico* analyses had not predicted TNPO3 as a target of miR-155, as well as with a greater stability or longer half-life of the protein that would require an extended period of time to reflect mRNA changes at the protein level.

### Silencing of selected HDFs in MDMs replicates the miR-155-induced phenotype

**Figure 10:**
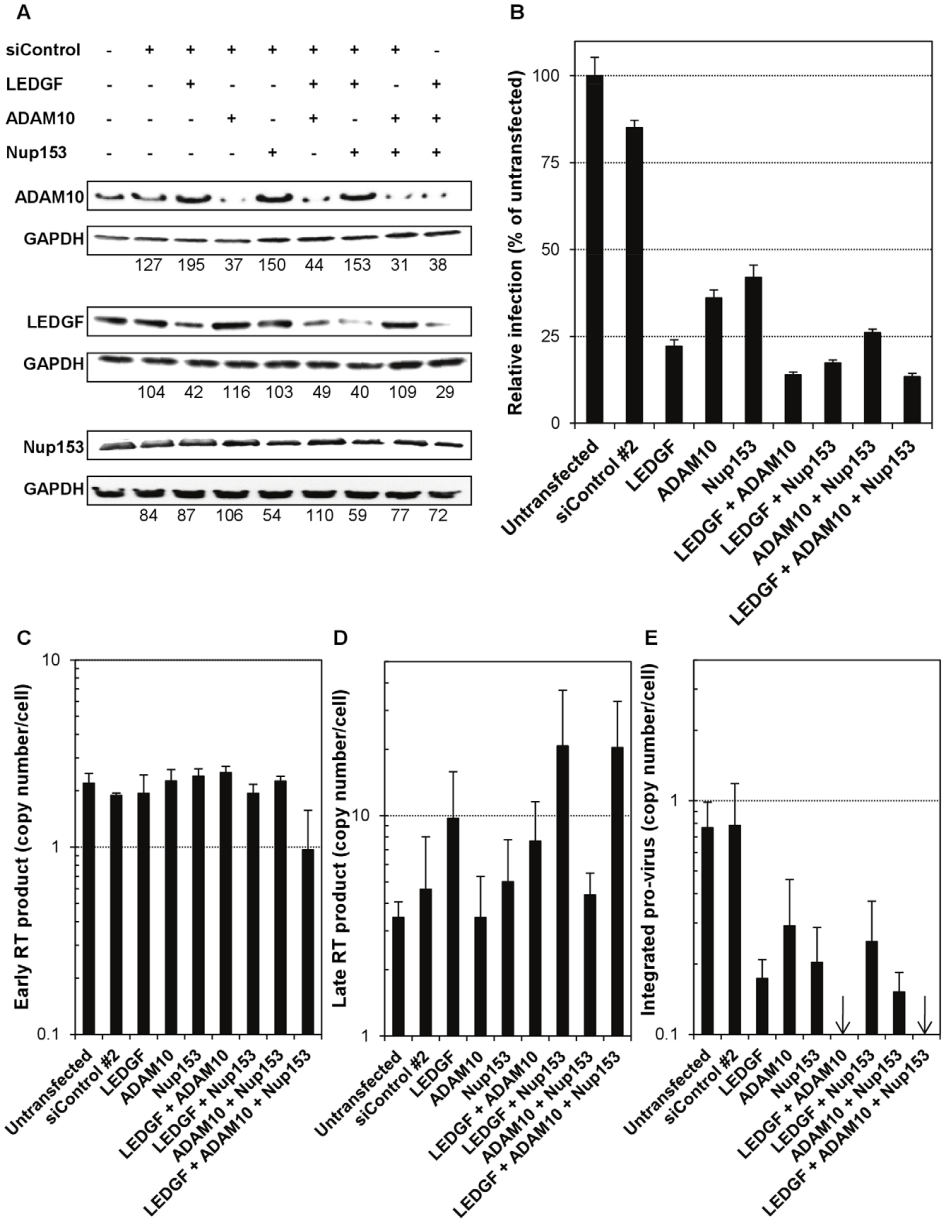
Silencing of selected HDFs replicate the miR-155-induced effects. MDMs from one donor were either untransfected or transfected for 48 h (LipoRNAiMax, Invitrogen) with Silencer Negative Control #2 siRNA (which has no significant sequence similarity to human gene sequences and is validated for use in human cells), or with Silencer Select siRNAs (two per target) for LEDGF, ADAM10 and Nup153 (all from Ambion), alone or in various combinations. Subsequently, cells were either lysed for western-blot analyses of protein levels (A) (as in Figure 9), or infected and lysed for quantitation of infection by luciferase activity (B), or infected and lysed for DNA isolation and quantitation of early (C) and late (D) viral RT products and integrated pro-virus (E) (as in Figure 6). Arrows indicate lack of amplification in real-time PCR, implying levels below the limit of detection of the technique.

Finally, in order to firmly demonstrate that the modulation of the levels of LEDGF, Nup153 and ADAM10 by miR-155 mediates the reduced susceptibility to infection in macrophages, through an accumulation of late RT products and absence or large reduction in integrated pro-virus, we performed a silencing experiment in which the expression of the above mentioned HDFs was reduced individually, or in various combinations, in a TLR- and miR-155-independent context, using Silencer Select siRNAs (Ambion). The siRNAs directed to each HDF of interest decreased its levels, whether applied individually or in combination, approximately to a similar extent (Figure 10A). Remarkably, the relative HDFs levels achieved, compared to those in untransfected MDMs, were similar to the levels obtained both in the context of TLR stimulation (relative to unstimulated) and ectopic expression of miR-155 (relative to untransfected) (Figure 9). Regarding infectivity, silencing of LEDGF decreased infection to a greater extent than silencing of ADAM10 or Nup153 (Figure 10B). Co-silencing of LEDGF with ADAM10 resulted in a slightly more robust reduction of infection than with Nup153, but both combinations were more efficient than silencing LEDGF alone, and co-silencing of ADAM10 and Nup153 also resulted in lower infectivity than silencing of each one individually. Finally, infectivity when co-silencing all three HDFs was not reduced further with respect to the co-silencing of LEDGF and ADAM10.

Subsequently, we performed quantitative real-time PCR to measure early and late RT products and integrated pro-virus, to determine if we could find the same pattern of viral cDNA observed in TLR3 stimulated and in miR-155 over-expressing MDMs. As shown in Figure 10C-E, no change was observed in detection of early RT products; however, we found that silencing of LEDGF led to some increase in the amount of late RT product, which was more robust in the co-silencing of LEDGF and Nup153 and when co-silencing all three HDFs. In addition, silencing of all HFDs led to a reduction in integrated pro-virus, becoming undetectable when co-silencing LEDGF and ADAM10 and when co-silencing all three HDFs (Figure 10E). In summary, co-depletion of LEDGF, ADAM10 and Nup153 replicated the effects, in terms of modulation of susceptibility to infection and viral DNA detection, observed through increased levels of miR-155 both in the context of TLR3 stimulation and ectopic expression in primary human MDMs.

## Discussion

Despite the success of combination anti-retroviral therapy and the potential use of anti-retroviral drugs for pre-exposure prophylaxis, there is a clear need for developing alternative interventions to prevent new HIV-1 infections. Macrophages are generally susceptible to HIV-1, although they contain restriction factors, such as tetherin [80]–[82] or SAMHD1 [83]–[88], that can restrict the efficiency of infection but are actively counteracted by viral proteins. In this context, further understanding of how otherwise susceptible cells may become naturally resistant to HIV-1, or at least display a substantial reduction in susceptibility to infection, is a topic of significant interest. The inhibitory effects of poly(I∶C) and LPS on HIV-1 infection in macrophages, through TLR3- and TLR4-mediated activation, respectively, have been previously described. LPS has been suggested to induce reduced expression of CD4 and CCR5, and to increase production of type I IFNs and release of β-chemokines that reduce infection by CCR5-using, HIV-1 envelope glycoproteins, among other mechanisms for its anti-HIV-1 effect [12]–[18]. Poly(I∶C) has been proposed to activate type I IFN-inducible antiviral factors (including APOBEC3G and tetherin), and to promote increased production of β-chemokines and higher levels of miRNAs that potentially target the 3′UTR of HIV-1 transcripts [23]. Wang et al. [24] recently proposed that stimulation of MDMs through TLR3, 4 and 7 reduces their susceptibility to HIV-1 infection and leads to the release of an unknown soluble factor that decreases infection of naïve MDMs. However, we wanted to further explore these effects and to compare them with the effects of macrophage stimulation through engagement of other relevant TLRs.

Using MDMs from multiple donors to minimize the effect of donor-to-donor variation, we found suppression or near suppression of infection in both single-round and multiple-round infections in TLR3- and TLR4-stimulated MDMs, but not in those stimulated through TLR2, TLR7 or TLR9, in which only a partial reduction of infection was observed. In addition, the same effects were also observed when infections were performed with a VSV-G-pseudotyped virus, suggesting that post-entry mechanisms of restriction of infection, rather than alterations at the level of viral entry, were mostly responsible for the observed inhibitory effects. This is in agreement with some previous studies that had also reported inhibition of infection of VSV-G-pseudotyped HIV-1 virions by TLR3 and TLR4 agonists (poly(I∶C) and LPS, respectively) in primary human myeloid cells [62], [63]. Furthermore, we found that only TLR2 stimulation reduced to some extent the expression levels of CD4 and CCR5, while all other ligands had no detectable effect by flow cytometry or immunofluorescence microscopy on receptor expression. Since infection with VSV-G-pseudotyped virions was not as sensitive to TLR2 stimulation as infection with BaL- or BR-pseudotyped viruses, it seems plausible that the effects on HIV-1 infectivity of MDMs through TLR2 stimulation may be more related to the modulation of the expression levels of CD4 and CCR5 than those induced through other TLRs.

We observed remarkable donor-to-donor variation in the production of type I IFNs into the culture supernatants upon TLR-stimulation of MDMs, with cells from some donors producing IFNα and/or IFNβ in response to all agonists investigated, some others only in response to selected ligands and some others not responding to any ligand. Furthermore, even though restricted expression of selected TLRs has been suggested (e.g., of TLR7 and 9 in plasmacytoid dendritic cells), we were able to detect expression of all TLRs investigated in MDMs from several donors. Moreover, we detected induction of gene expression of type I IFNs among poly(I∶C)-, LPS- and Imiquimod-stimulated MDMs, although with some donor-to-donor variation as well, further supporting the notion that MDMs contained functional receptors and responded to the various TLR ligands. Thus, it seems that production of type I IFNs may not play a major role in the differences observed in reduction of susceptibility to HIV-1 infection across TLR-stimulated MDMs. This appears to be in contrast to some previous studies. For example, a role for type I IFNs in poly(I∶C)-induced, TLR3-mediated, and in LPS-induced, TLR4-mediated anti-HIV-1 effects in macrophages has been suggested, although in the presence of IFNα and/or IFNβ concentrations in culture supernatants in the low pg/ml range [23], [24]. In addition, a previous study that proposed a partial role for type I IFNs in the anti-HIV-1 effect of LPS in macrophages was actually performed in the context of an established infection and via exogenous treatment, and did not actually measure type I IFNs produced in response to LPS [12]. Therefore, we believe that our experiments reflect the variability that can be expected from a large group of normal human donors, which is not a factor in studies performed with mouse models or with human or murine cell lines.

In addition, all TLR-stimulated MDMs produced high amounts of pro-inflammatory cytokines TNF-α and IL-6, and of β-chemokines CCL3–5, indicating as well that they did respond robustly to stimulation, and we did not find any noticeable difference that could correlate with the suppression vs. reduction of infection observed with the various TLR ligands. Remarkably, culture supernatants from poly(I∶C)-, LPS- and Imiquimod-treated MDMs from two donors did not differ in their ability to reduce susceptibility to HIV-1 infection when transferred to naïve MDMs, suggesting that indeed there is a contribution to anti-HIV-1 effects of soluble factor(s) released from MDMs upon TLR stimulation, but this contribution seems to be similar for TLR3, TLR4 and TLR7, as recently suggested by Wang et al. [24]. However, these passive transfer experiments never led to a complete suppression of infection, resulting in partial reductions of, at most, a similar magnitude to those observed through TLR7 or TLR9 stimulation. Therefore, the mechanism(s) for the suppression vs. partial reduction of infection upon TLR3/4 and TLR7/9 engagement, respectively, remained unclear.

Since macrophage stimulation through TLRs has also been shown to modulate miRNAs, we investigated whether potential differences in miRNA expression among MDMs stimulated with poly(I∶C), LPS and Imiquimod might contribute to the differential anti-HIV-1 effect. We found that miR-155 was consistently induced in MDMs from multiple donors by poly(I∶C) and LPS but not by Imiquimod, and that the induction through TLR3 was significantly stronger than through TLR4. Induction of miR-155 in the human monocytic cell line THP1 stimulated with LPS [28] and in murine bone marrow-derived macrophages stimulated with poly(I∶C) [27] has been reported, but, to our knowledge, this is the first report of TLR3-mediated induction of miR-155 expression in primary human macrophages. Although a recent study by Zhou et al. [89] has shown increased levels of miR-155 through TLR7 engagement in human plasmacytoid dendritic cells, we did not observe a similar induction in primary human macrophages, and no study has yet reported a stimulatory effect of TLR7 engagement on miR-155 expression in macrophages.

Individual TLRs can trigger common as well as specific biological responses. The main Toll-interleukin 1 receptor (TIR) domain-containing adaptor, myeloid differentiation primary response gene 88 (MyD88), is recruited by all TLRs except TLR3, while TIR domain-containing adaptor-inducing IFNβ (TRIF) is used by TLR3 and TLR4 [5]. Both MyD88-dependent and TRIF-dependent pathways have been shown to result in NF-κB-mediated, miR-155 induction in murine macrophages [27], although it has not been reported to occur upon TLR7 stimulation. However, the requirements for miR-155 induction in human macrophages may be different and its absence in TLR7-stimulated MDMs may not be attributed to a lack of receptor expression or stimulation, as shown above. Regarding a potential role of the cytosolic receptors of dsRNA retinoic acid inducible gene-I and melanoma differentiation-associated gene 5 (RIG-I and MDA-5, respectively) in the poly(I∶C)-induced effects reported in our studies, it is generally accepted that naked poly(I∶C) added to cells in the culture medium is more likely to induce downstream signaling events through interaction with TLR3 in an endosomal localization, than through interaction with RIG-I or MDA-5, which are specialized in sensing cytoplasmic dsRNA. Cytosolic localization of poly(I∶C) seems to require a transfection step to bypass endocytosis and to reach the cytoplasm of the cell, or the addition to the cells of extremely high concentrations of naked poly(I∶C) [90]–[96]. Once in the cytoplasm, low molecular weight poly(I∶C) and certain RNA structures seem to determine RIG-I specificity, while MDA-5 appears to recognize high molecular weight poly(I∶C) and specific dsRNAs (reviewed in [97]). Therefore, since our studies were performed with a high molecular weight poly(I∶C), we can't exclude that MDA-5 may have contributed to the reported effects, but it seems more likely that they are primarily mediated through TLR3.

More importantly, we functionally demonstrated the contribution of miR-155 to the suppression of HIV-1 infection in poly(I∶C)-treated MDMs, by partially reverting this anti-HIV-1 effect through inhibition of miR-155 with a specific antagomir. As expected, the anti-miR-155 antagomir abrogated the poly(I∶C)-induced miR-155 increase, and this resulted in a significantly higher susceptibility to infection, which became more similar to that observed in TLR7-stimulated MDMs that do not display increased miR-155 levels. Further confirmation of the anti-HIV-1 effects of miR-155 was obtained through ectopic expression in both primary MDMs and human cell lines. MDMs from multiple donors showed a significant reduction in infection after transfection with a pre-miR-155-expressing plasmid. In both contexts, we found a statistically significant negative relationship between relative fold increase in miR-155 levels and the level of relative infection (compared to unstimulated or untransfected controls). Similarly, HOS and U87 cells transfected for miR-155 expression displayed a negative correlation between fold-increase in miR-155 levels and susceptibility to infection with both BaL- and VSV-G-pseudotyped viruses, again pointing to the involvement of post-entry mechanism(s) in the anti-HIV-1 effects of miR-155.

We subsequently performed real time PCR for detection and quantitation of viral DNA forms (early and late RT products, 2-LTR circles and integrated pro-viruses). These experiments demonstrated an accumulation of late RT products in cells expressing higher levels of miR-155, with either undetectable or very low levels of 2-LTR circles and integrated pro-viral forms of viral DNA, and unaltered levels of early RT products. This phenotype was consistently observed both in the context of ectopic expression of miR-155 in MDMs from multiple donors and cell lines (but not in scrambled control-transfected cells), and in TLR3-stimulated MDMs from multiple donors. In addition, anti-miR-155 antagomir (but not the scrambled antagomir control) reverted the viral DNA profile induced by poly(I∶C) stimulation, making it identical to that observed in unstimulated and Imiquimod-stimulated MDMs. These results confirmed that entry and early post-entry events in the viral life cycle (i.e., initiation of reverse transcription) are not affected in cells with high levels of miR-155, since there is no difference in generation of early RT products, which has been reported as the main anti-HIV-1 effect of IFNα on macrophages [98], [99]. Moreover, the absence or near absence of 2-LTR circles and integrated pro-viruses, together with the remarkable accumulation of late RT products, seem to indicate that, despite completion of reverse transcription, the ability to enter the nucleus of PICs in infected cells is abrogated or severely compromised due to the high levels of miR-155, suggesting potential alterations in the cellular trafficking mechanisms utilized by the virus for nuclear import of PICs.

It should be noted that we generally detected higher amounts of late than early RT products, not only with poly(I∶C) stimulation and ectopic expression of miR-155, but also in unstimulated and untransfected cells, albeit to a much lower extent. Although previously reported in the literature [100], we believe that this slightly higher abundance of late versus early RT products may be related with the fact that our studies were performed in primary macrophages (in which reverse transcription is expected to be less efficient than in continuously dividing cell lines or other cell types such as T cells), with a single-round infectious virus, and we only evaluated the abundance of viral cDNA forms at 48 hours post-infection. Therefore, it is possible that most of the early RT products that could have been detected at earlier time points were actually able to proceed through reverse transcription to become late products, which then tend to accumulate, especially in those conditions in which nuclear import seems to be impaired (poly(I∶C) stimulation and ectopic expression of miR-155). Future kinetic studies will investigate in more detail the abundance of the various viral cDNA forms and their stability over time in different experimental conditions.

**Figure 11:**
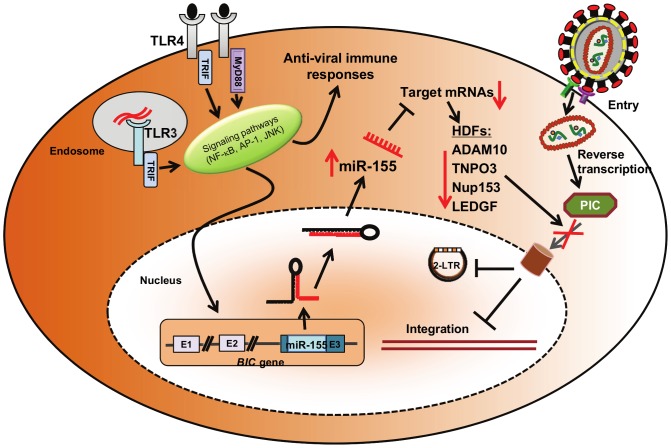
Schematic representation of potential miR-155 effects on HIV-1 dependency factors (HDFs). We found that increased levels of miR-155 are induced in macrophages by TLR3 and TLR4 stimulation, and that at least in the case of TLR3, miR-155 contributes to the induced anti-HIV-1 effect. In addition, increased levels of miR-155 in MDMs lead to consistent decreases in the mRNA and protein levels of ADAM10, TNPO3, Nup153 and LEDGF/p75 (PSIP1), which are all HDFs known to participate in trafficking and/or nuclear import of pre-integration complexes (PICs) during HIV-1 infection. These findings, together with our results on the accumulation of late reverse transcription products and the absence or large reduction of integrated pro-viruses and 2-LTR circles in cells expressing increased miR-155 levels, lead us to hypothesize that it is possible a model in which a miR-155 action upon expression of multiple targets, rather than a single one, might generate a cellular environment that is less conducive to HIV-1 infection and thus mediate the anti-HIV-1 effects of miR-155.

Using the information from large-scale RNA interference screens and the growing body of scientific literature available on HDFs [74], [75], [101]–[103], we performed *in silico* target prediction analyses and identified several confirmed and possible HDFs that could potentially be targeted by miR-155 and therefore might be involved in the observed phenotype. Since it has been proposed that mRNA levels tend to closely reflect the impact of miRNAs on gene expression [104], we performed relative quantitation of mRNA levels of predicted miR-155 targets and of some other confirmed and possible HDFs that could be important for the phenotypic alteration induced by higher miR-155 levels. We found that both ectopic expression of miR-155 in MDMs from multiple donors, and poly(I∶C) and LPS stimulation in MDMs from two donors, led to consistent decreases in the mRNA levels of ADAM10, TNPO3, NUP153 and LEDGF/p75 (PSIP1), giving credence to a model in which a combined action upon expression of multiple targets, rather than a single one, might mediate the reported anti-HIV-1 effects of miR-155 (Figure 11). In addition, using 3′UTR target reporter assays, we validated that the 3′UTRs of all four factors contain target sites for miR-155, although the magnitude of the inhibition of expression differed among them. These differences in magnitude actually were paralleled by the reduction observed in the protein levels of LEDGF, ADAM10, Nup153 and TNPO3 in MDMs with higher miR-155 levels, achieved either through poly(I∶C)- or LPS-stimulation, or through ectopic expression. Altogether, these results demonstrate that miR-155 targets several HDFs involved in trafficking and/or nuclear import of PICs, and that the combination of these effects seems to result in the potent anti-HIV-1 activity reported in this study.

ADAM10, a metalloprotease that was identified as HDF in a functional genomic screen [101], has been recently proposed to be required for trafficking or nuclear import of PICs [105]. Although completion of reverse transcription was not affected by depletion of ADAM10, an accumulation of late RT products was not observed and only a modest decrease (2–3 fold) in the amount of integrated pro-viruses was reported [105]. Multiple studies have shown that TNPO3 is required for full infectivity of HIV-1, and effects at the level of pre- and post-nuclear import of PICs have been reported upon its depletion [106]–[108]. Nup153 is a nuclear pore complex protein also reported to mediate nuclear import of HIV-1 PICs [109]–[111]. However, Nup153 knock-down resulted in a modest reduction in 2-LTR circles and in a large decrease in integrated pro-viruses, while the levels of late reverse transcription products were unaffected [110], probably suggesting a combination of pre- and post-nuclear import effects. LEDGF/p75, known to mediate tethering of the HIV-1 pro-viral DNA to the host chromatin, has also been proposed to participate in nuclear targeting of PICs (reviewed by Llano et al. [112]). LEDGF/p75-depleted cells display a ten-fold or greater decrease in the amount of integrated pro-viral DNA, but concurrently with a two-fold increase in the amount of 2-LTR circles [113], making this phenotype also different from that induced by miR-155. Interestingly, Matreyek and Engelman have reported that co-depletion of Nup153 and TNPO3 yielded synergistic inhibitory effects on HIV-1 infection [110], underscoring the fact that simultaneous targeting of multiple HDFs might be a potentially useful therapeutic/preventive approach.

In our studies, the absence or greatly reduced detection of integrated pro-viruses and 2-LTR circles suggests that no or very little amount of viral DNA is actually imported into the nucleus, and this would then correlate with the observed accumulation of late RT products. Unlike the 2-LTR circles, degradation of late RT products might be prevented, since it has been reported that PICs protect viral DNA from degradation [114]. Therefore, it is plausible that a limited availability of one or more cellular proteins involved in the trafficking or nuclear import of PICs within the infected cell may result in the absence of nuclear import and accumulation of late RT products in TLR3-stimulated and miR-155 over-expressing MDMs. This was further confirmed by the fact that, independently of TLR stimulation or higher miR-155 levels, co-depletion in MDMs of LEDGF with ADAM10 or Nup153, or of all three HDFs at the same time (as obtained in the context of higher expression of miR-155), resulted as well in an accumulation of late RT products and in a large reduction (even to below the levels of detection) in the amount of integrated pro-virus. However, since it has been recently suggested that the reduced protein output induced by miRNAs may first be due to translational repression, subsequently followed by mRNA degradation [115], [116], a potential contribution to the effects described above in the context of TLR stimulation or over-expression of miR-155, of other miR-155 potential targets such as importins α3 and α5 (KPNA4 and 1, respectively, for which the mRNA levels were not as consistently decreased by higher miR-155, despite being strongly predicted *in silico* to be miR-155 targets), or some other unidentified HDF, may not yet be completely excluded and deserves further investigation. Overall, our findings demonstrate a novel contribution of miR-155 to the poly(I∶C)-induced anti-HIV-1 effects in primary macrophages, which may help in the characterization of known and/or yet undefined host factors that play important roles in the HIV-1 life cycle, and could potentially lead to innovative preventive or therapeutic anti-HIV-1 strategies.

## Supporting Information

Figure S1 **TLR stimulation does not alter CD4 and CCR5 expression in primary macrophages.** MDMs were cultured overnight in the absence (A) or in the presence of specific ligands for TLR3 (B), TLR4 (C) or TLR7 (D), and then stained with goat anti-human CD4 (AF-379-NA, R&D Systems) and mouse anti-human CCR5 (CTC8, R&D Systems) Abs, followed by Alexa Fluor 488-conjugated anti-goat IgG and Alexa Fluor 594-conjugated anti-mouse IgG Abs, respectively. DAPI (4–6′-diamidino-2-phenylindole) was used for staining of nuclei. Images were obtained using an Olympus 1×81 deconvolution fluorescent microscope and SlideBook 5.0 software (Intelligent Imaging Innovations, Inc.). No noticeable differences in CD4 or CCR5 expression were observed between unstimulated or ligand-stimulated MDMs. (E) MDMs cultured for 16 h either unstimulated (red) or with the ligands for TLR2 (blue), TLR3 (orange), TLR4 (light green), or TLR7 (dark green), were collected and stained with FITC-conjugated anti-CD4 and PE-conjugated anti-CCR5 Abs (eBioscience), or with the appropriate isotype control Abs. Data were collected using a BD FACSCalibur flow cytometer with CellQuest software and analyzed using FlowJo flow cytometry analysis software; unstimulated cells stained with an isotype control antibody are shown in grey.
(TIF)

Figure S2 **TLR expression in MDMs.** Cells from several donors were collected after differentiation and stained with PE-conjugated anti-TLRs (solid line) or the appropriate PE-conjugated isotype controls (shown in grey) antibodies. Data were collected using a BD FACSCalibur flow cytometer with CellQuest software and analyzed using FlowJo flow cytometry analysis software. Results from two representative donors are shown.
(TIF)

Figure S3 **Type I IFN gene expression in primary macrophages upon TLR stimulation.** Total RNA was isolated from MDMs from four different donors that were cultured for 16 h unstimulated or with poly(I∶C), LPS or Imiquimod, and in the case of poly(I∶C), they had been either untransfected or transfected (prior to stimulation) with anti-miR-155 or anti-miR-scr. Subsequently, we performed relative quantitation of mRNAs by real-time RT-PCR of type I IFNs (IFNα1, IFNα2 and IFNβ) using TaqMan Gene Expression assays (Applied Biosystems). Results were calculated using 18S rRNA as internal control and are shown as fold-change (mean ± SD from two independent quantitations) with respect to the unstimulated control.
(TIF)

Figure S4 **Anti-HIV-1 effect of supernatants from TLR-stimulated MDMs.** MDMs from one donor were cultured for 16 h unstimulated or with poly(I∶C), LPS or Imiquimod, and then supernatants were collected, clarified, aliquoted and stored at −80°C until use. Fresh “naïve” macrophages from two different donors were treated for 2 or 6 h with the conditioned media, and then washed twice with PBS and infected with BAL pseudotypes for 48 hours. Cells were then lysed and processed for luciferase activity, and results are shown as actual luc activity in cell lysates measured as relative light units per second (mean ± SD of experiments performed in quadruplicate).
(TIF)

Figure S5 **Effects of TLR stimulation on miRNA expression profiles in primary macrophages.** miRNA expression profiles were studied in RNA isolated from MDMs from one donor, either unstimulated or stimulated for 16 hours with the ligands for TLR3 (10 µg/ml poly(I∶C)), TLR4 (10 µg/ml LPS), or TLR7 (5 µg/ml Imiquimod), using GenoExplorer microRNA chips (GenoSensor). Background-subtracted data was first normalized against the average of the positive controls, and then used to calculate differences in expression between stimulated and unstimulated cells, and the statistical analysis of those differences by using a Student's t test to compare normalized signal intensity for each miRNA in replicate measurements of stimulated and unstimulated cells. Altered expression was considered significant when greater than 2-fold change and an associated *p* value ≤0.01. (A) Venn diagram showing the up-regulated miRNAs in TLR3-, TLR4- and TLR7-stimulated MDMs; (B) List of miRNAs with their fold change versus unstimulated.
(TIF)

Figure S6 **Regression analysis of miR-155 levels and percentage of infection in MDMs.** (A) Using all infection and miR-155 data from poly(I∶C)-, LPS- and Imiquimod-stimulated MDMs, we performed curve estimation using a logarithmic regression model with miR-155 levels as independent variable and relative infection data as dependent variable (SPSS), and found that the model is a good fit for the data (R^2^ = 0.48, p<0.001), which suggests that susceptibility to infection in TLR3-, TLR4- and TLR7-stimulated MDMs is determined, at least in part, by miR-155 levels. (B) In the context of ectopic expression of miR-155 in MDMs from multiple donors, curve estimation using a logarithmic regression model of miR-155 levels (independent variable) and relative infection data (dependent variable), indicated a good fit of the model (R^2^ = 0.514, p<0.01); however, careful evaluation of the data revealed the presence of an outlier that resulted in a moderate multiple correlation coefficient (R = 0.717). Removal of the outlier (circled in red) increased even further the goodness of the model (R^2^ = 0.804, p<0.001), suggesting that susceptibility to infection in miR-155-transfected MDMs is determined, in large part, by miR-155 levels.
(TIF)

Figure S7 **Efficiency of transfection of MDMs with an antagomir control.** Primary human MDMs were untransfected (A,B), or transfected with the Ambion Cy3 dye-labeled Anti-miR scrambled negative control (anti-miR-scr, which does not target any known human miRNA) (Applied Biosystems), at an optimized concentration of 30 nM using Lipofectamine RNAiMax transfection reagent (Invitrogen) (C,D). At 24 h post-transfection, MDMs were washed with PBS and replaced with media, and bright field (A,C) and fluorescent (B,D) images (10× magnification) were obtained using an Olympus 1×81 deconvolution fluorescent microscope and SlideBook 5.0 software. The extent of Cy3 signal indicates a good transfection efficiency of primary MDMs.
(TIF)

Figure S8 **Efficiency of transfection of MDMs with plasmid DNA.** Primary human MDMs were untransfected (A,B), or transfected with the GFP-encoding, miR-155 expression plasmid (pEZX-miR-155) (C,D) or scrambled control (pEZX-scrambled) (E,F) (GeneCopoeia), using jetPEI-Macrophage transfection reagent (Polyplus), following manufacturer's instructions. After 48 h, bright field (A,C,E) and fluorescent (B,D,F) images (10× magnification) were obtained using an Olympus 1×81 deconvolution fluorescent microscope and SlideBook 5.0 software (Intelligent Imaging Innovations, Inc.). The extent of GFP expression indicates a good transfection efficiency of primary MDMs.
(TIF)

Figure S9 **Detection of 2-LTR circles in MDMs.** Total DNA was isolated at 48 h post-infection from BaL pseudotype-infected MDMs (shown from an individual donor) that were unstimulated, or stimulated with poly(I∶C), LPS or Imiquimod, or treated with two different concentrations of the integrase inhibitor Raltegravir (RAL). Real-time PCR was performed in total DNA for the relative quantitation of 2-LTR circles, as described in Materials and Methods. Results were calculated with respect to CCR5 (used as endogenous control to normalize for DNA recovery and number of cells), and are shown relative to the amounts detected in unstimulated cells, as mean ± SD from two independent quantitations.
(TIF)

Table S1 **Primers and probes used for real-time PCR analysis of viral DNA products.**
(DOCX)

Table S2 **Modulation of HIV-1 infection and miR-155 levels in MDMs with TLR stimulation (n = 10–12).**
(DOCX)

Table S3 **Statistical analysis of miR-155 levels in TLR-stimulated and unstimulated MDMs.**
(DOCX)
