# Supplementary figures and images for: A Role for microRNA-155 Modulation in the Anti-HIV-1 Effects of Toll-Like Receptor 3 Stimulation in Macrophages
Source: PLoS Pathog. 2012 Sep 20;8(9):e1002937. doi: 10.1371/journal.ppat.1002937 (PMC3447756; doi:10.1371/journal.ppat.1002937)

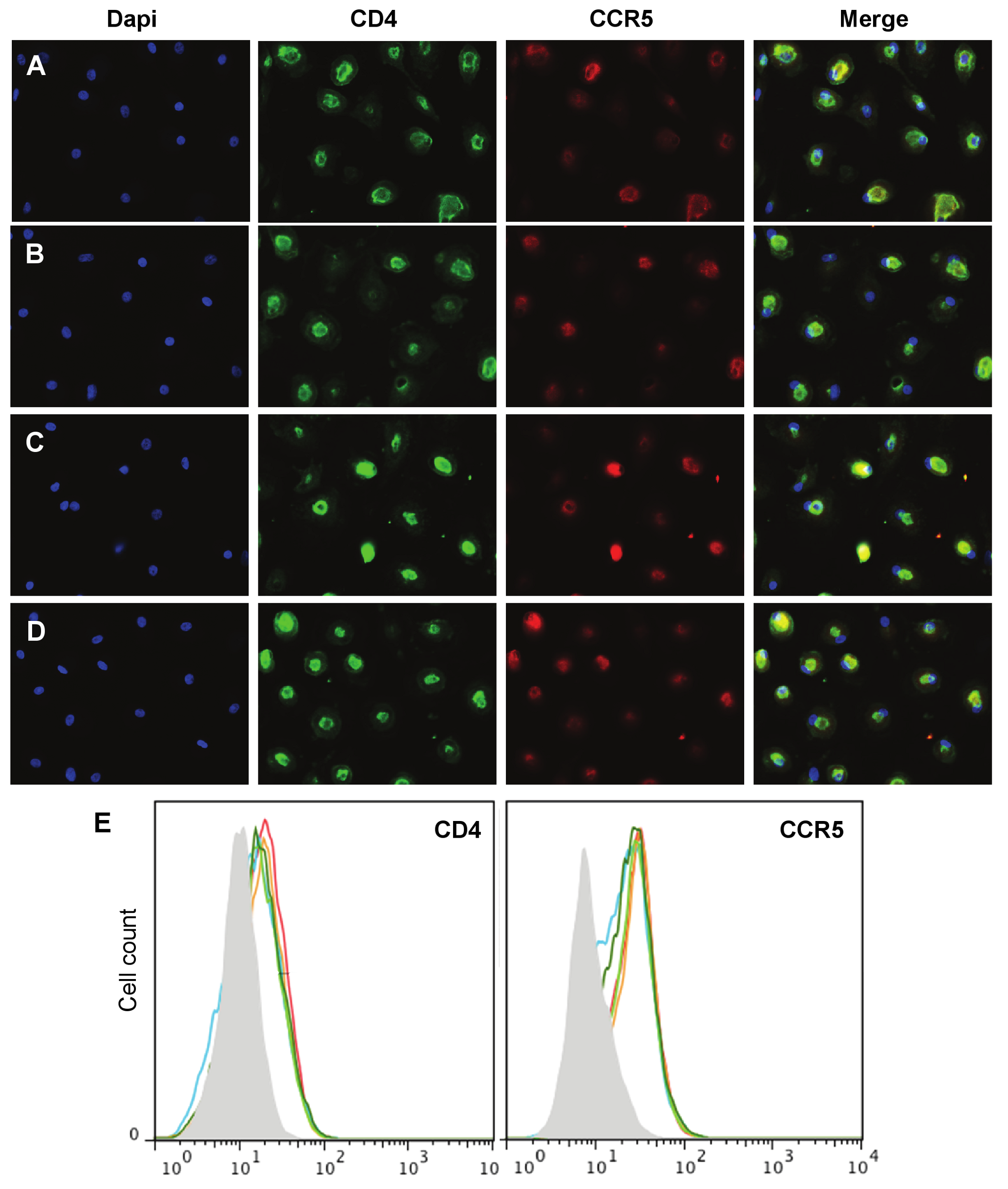

Supplement: Figure S1 — TLR stimulation does not alter CD4 and CCR5 expression in primary macrophages. MDMs were cultured overnight in the absence (A) or in the presence of specific ligands for TLR3 (B), TLR4 (C) or TLR7 (D), and then stained with goat anti-human CD4 (AF-379-NA, R&D Systems) and mouse anti-human CCR5 (CTC8, R&D Systems) Abs, followed by Alexa Fluor 488-conjugated anti-goat IgG and Alexa Fluor 594-conjugated anti-mouse IgG Abs, respectively. DAPI (4–6′-diamidino-2-phenylindole) was used for staining of nuclei. Images were obtained using an Olympus 1×81 deconvolution fluorescent microscope and SlideBook 5.0 software (Intelligent Imaging Innovations, Inc.). No noticeable differences in CD4 or CCR5 expression were observed between unstimulated or ligand-stimulated MDMs. (E) MDMs cultured for 16 h either unstimulated (red) or with the ligands for TLR2 (blue), TLR3 (orange), TLR4 (light green), or TLR7 (dark green), were collected and stained with FITC-conjugated anti-CD4 and PE-conjugated anti-CCR5 Abs (eBioscience), or with the appropriate isotype control Abs. Data were collected using a BD FACSCalibur flow cytometer with CellQuest software and analyzed using FlowJo flow cytometry analysis software; unstimulated cells stained with an isotype control antibody are shown in grey. (TIF) [file ppat.1002937.s001.tif]

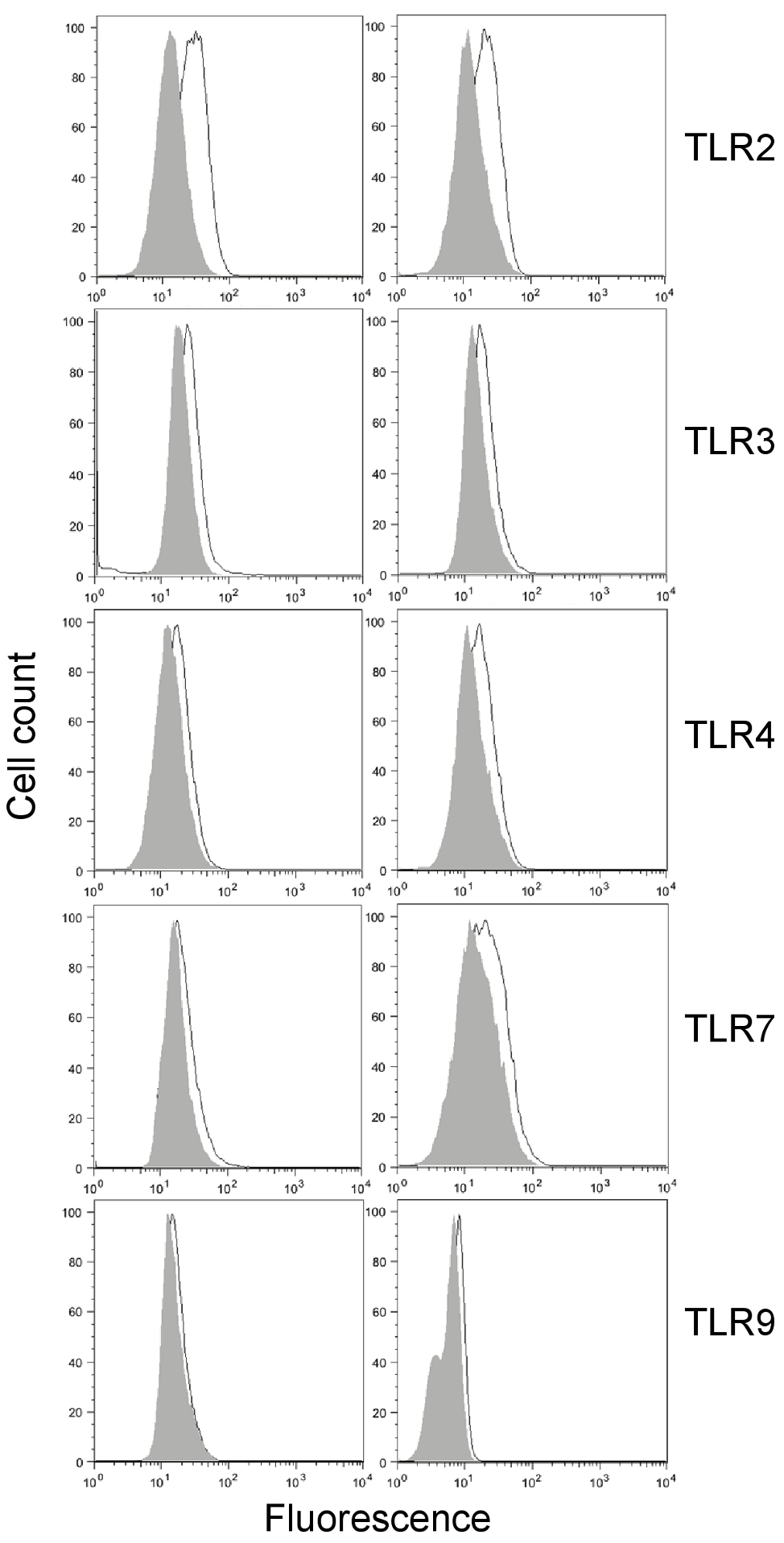

Supplement: Figure S2 — TLR expression in MDMs. Cells from several donors were collected after differentiation and stained with PE-conjugated anti-TLRs (solid line) or the appropriate PE-conjugated isotype controls (shown in grey) antibodies. Data were collected using a BD FACSCalibur flow cytometer with CellQuest software and analyzed using FlowJo flow cytometry analysis software. Results from two representative donors are shown. (TIF) [file ppat.1002937.s002.tif]

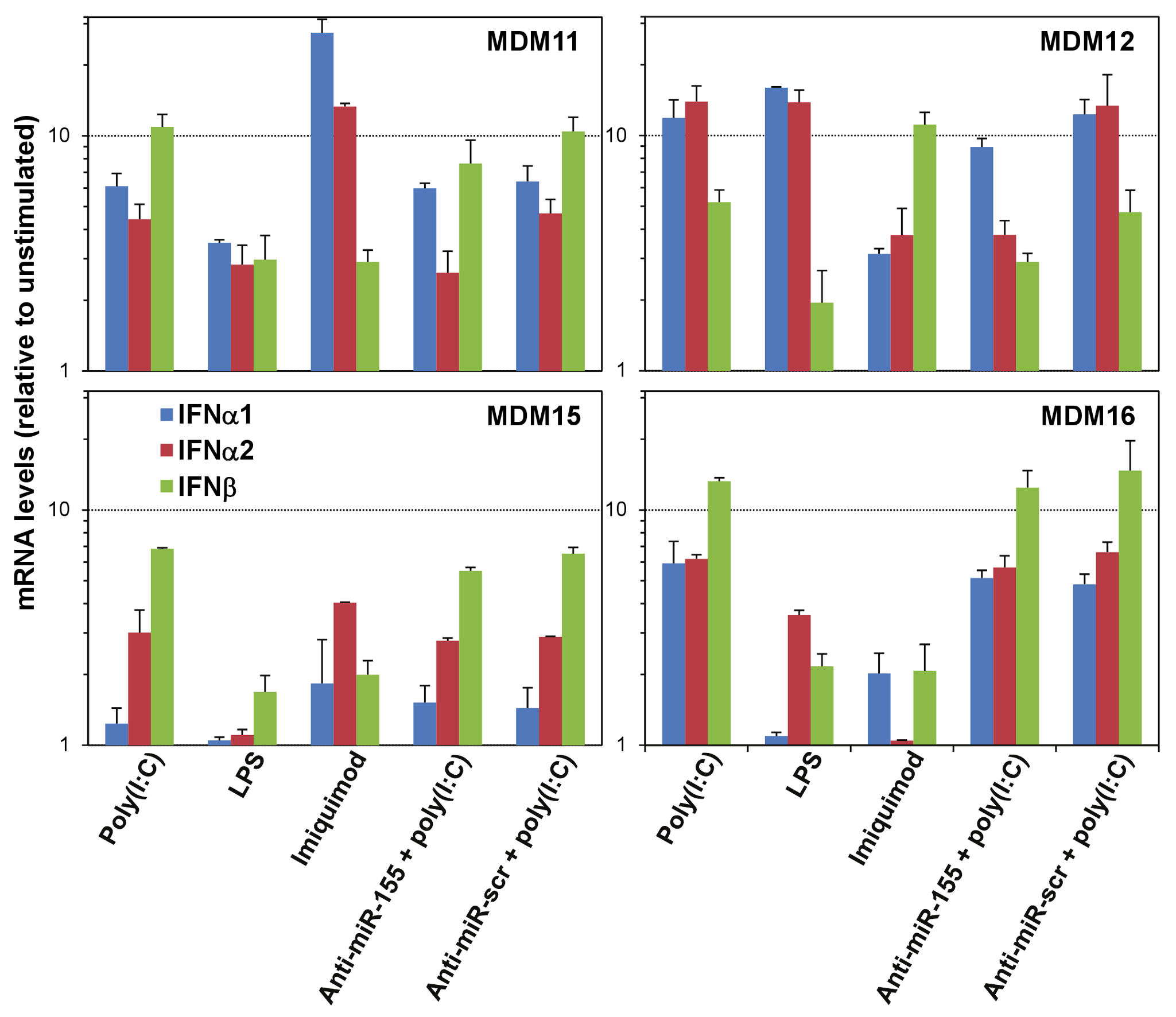

Supplement: Figure S3 — Type I IFN gene expression in primary macrophages upon TLR stimulation. Total RNA was isolated from MDMs from four different donors that were cultured for 16 h unstimulated or with poly(I∶C), LPS or Imiquimod, and in the case of poly(I∶C), they had been either untransfected or transfected (prior to stimulation) with anti-miR-155 or anti-miR-scr. Subsequently, we performed relative quantitation of mRNAs by real-time RT-PCR of type I IFNs (IFNα1, IFNα2 and IFNβ) using TaqMan Gene Expression assays (Applied Biosystems). Results were calculated using 18S rRNA as internal control and are shown as fold-change (mean ± SD from two independent quantitations) with respect to the unstimulated control. (TIF) [file ppat.1002937.s003.tif]

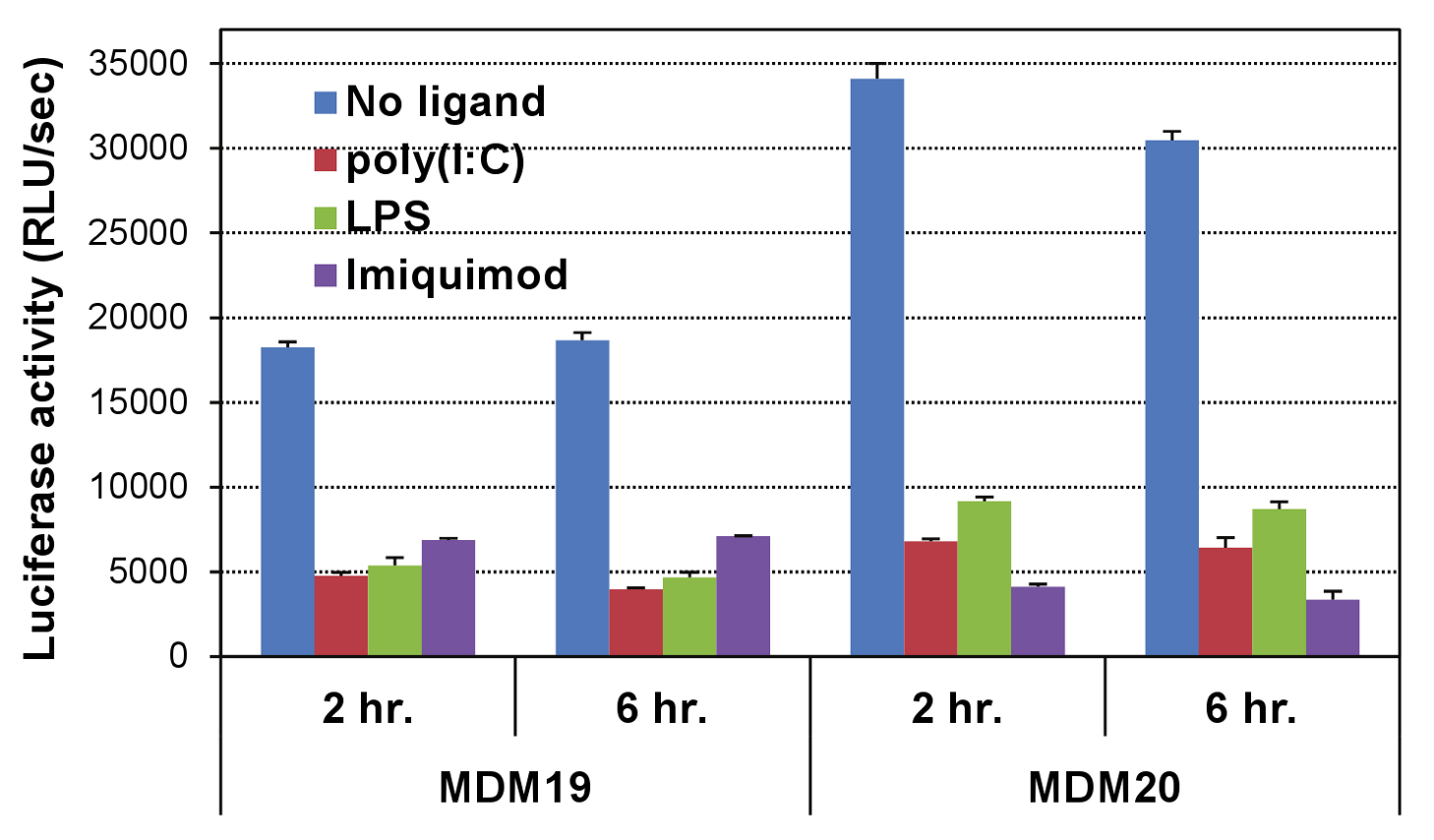

Supplement: Figure S4 — Anti-HIV-1 effect of supernatants from TLR-stimulated MDMs. MDMs from one donor were cultured for 16 h unstimulated or with poly(I∶C), LPS or Imiquimod, and then supernatants were collected, clarified, aliquoted and stored at −80°C until use. Fresh “naïve” macrophages from two different donors were treated for 2 or 6 h with the conditioned media, and then washed twice with PBS and infected with BAL pseudotypes for 48 hours. Cells were then lysed and processed for luciferase activity, and results are shown as actual luc activity in cell lysates measured as relative light units per second (mean ± SD of experiments performed in quadruplicate). (TIF) [file ppat.1002937.s004.tif]

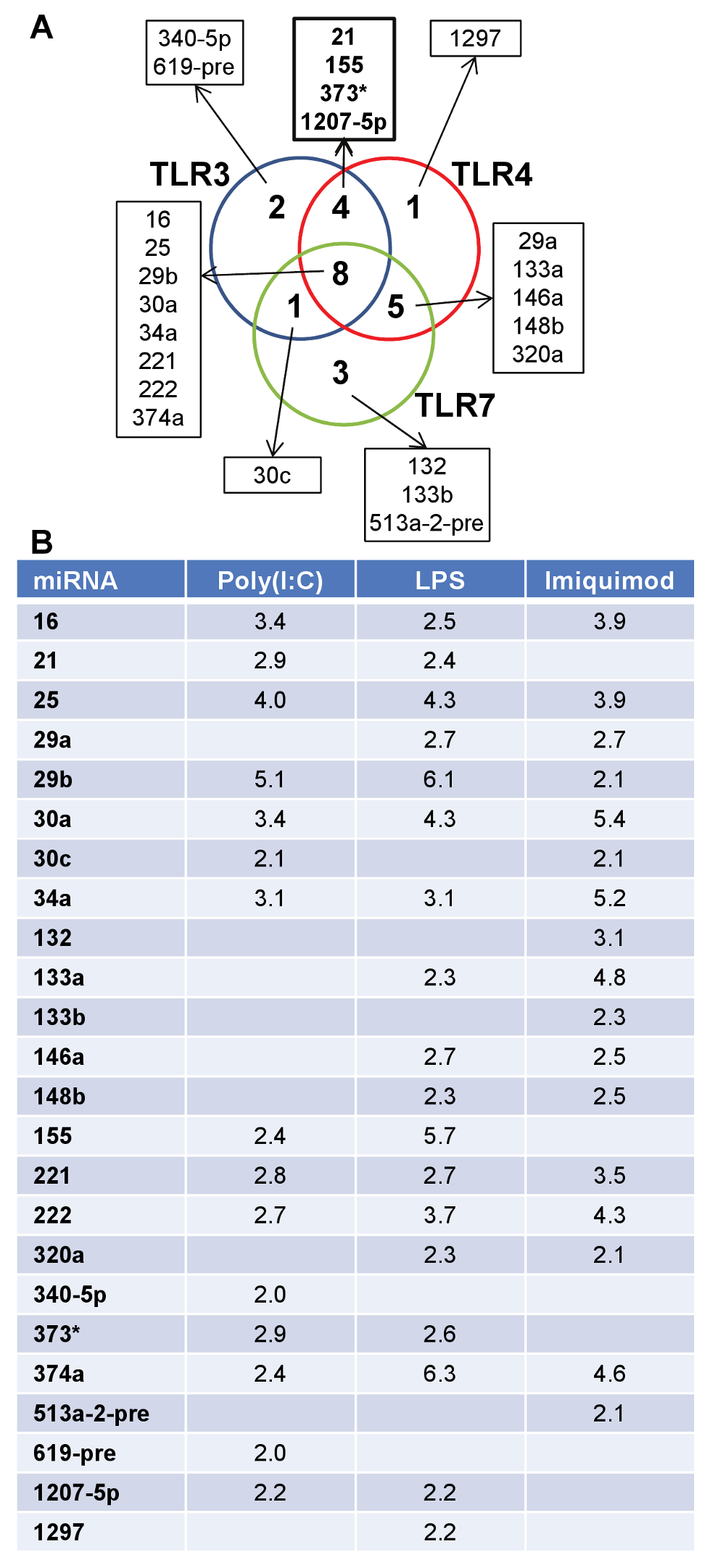

Supplement: Figure S5 — Effects of TLR stimulation on miRNA expression profiles in primary macrophages. miRNA expression profiles were studied in RNA isolated from MDMs from one donor, either unstimulated or stimulated for 16 hours with the ligands for TLR3 (10 µg/ml poly(I∶C)), TLR4 (10 µg/ml LPS), or TLR7 (5 µg/ml Imiquimod), using GenoExplorer microRNA chips (GenoSensor). Background-subtracted data was first normalized against the average of the positive controls, and then used to calculate differences in expression between stimulated and unstimulated cells, and the statistical analysis of those differences by using a Student's t test to compare normalized signal intensity for each miRNA in replicate measurements of stimulated and unstimulated cells. Altered expression was considered significant when greater than 2-fold change and an associated p value ≤0.01. (A) Venn diagram showing the up-regulated miRNAs in TLR3-, TLR4- and TLR7-stimulated MDMs; (B) List of miRNAs with their fold change versus unstimulated. (TIF) [file ppat.1002937.s005.tif]

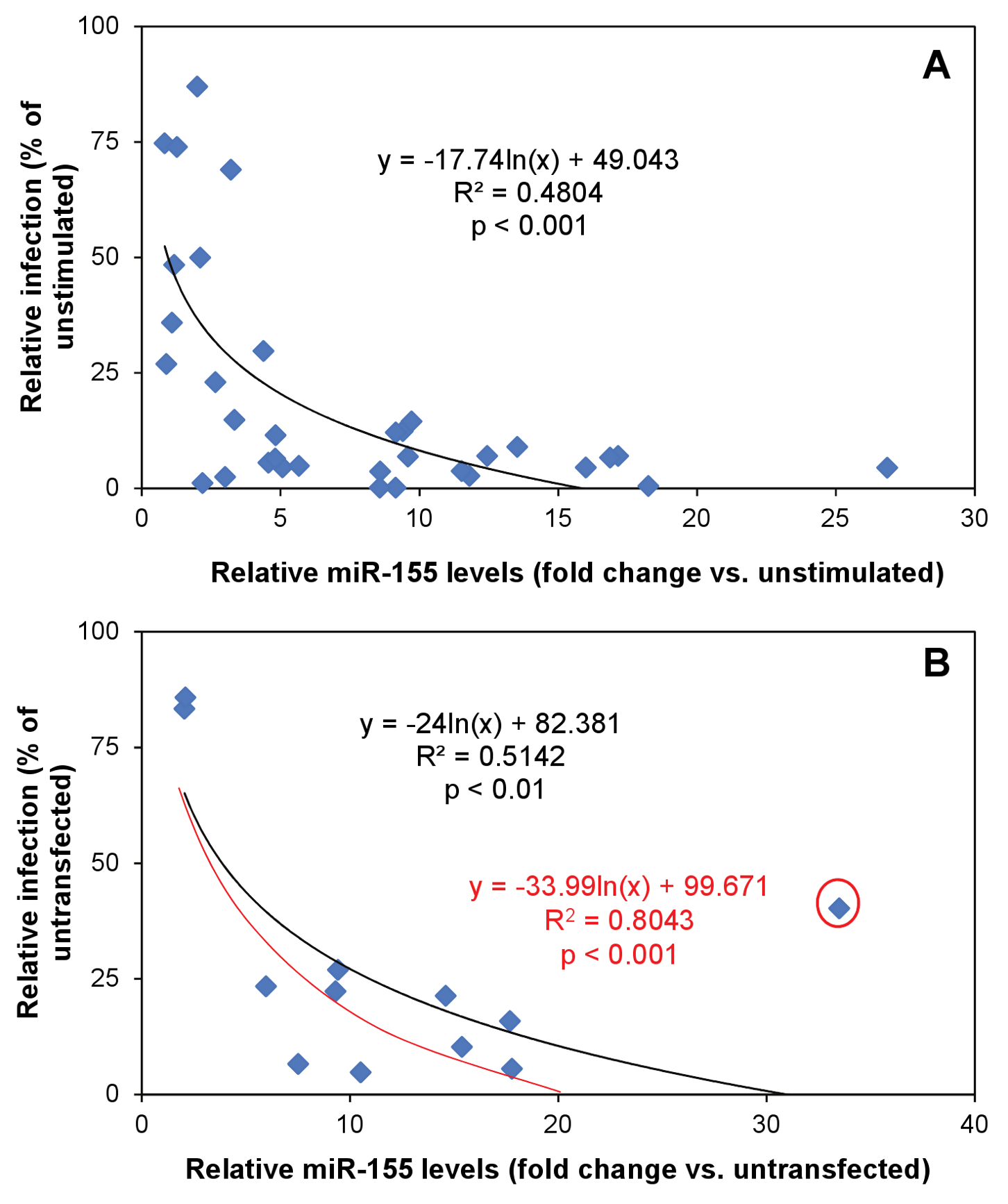

Supplement: Figure S6 — Regression analysis of miR-155 levels and percentage of infection in MDMs. (A) Using all infection and miR-155 data from poly(I∶C)-, LPS- and Imiquimod-stimulated MDMs, we performed curve estimation using a logarithmic regression model with miR-155 levels as independent variable and relative infection data as dependent variable (SPSS), and found that the model is a good fit for the data (R2 = 0.48, p<0.001), which suggests that susceptibility to infection in TLR3-, TLR4- and TLR7-stimulated MDMs is determined, at least in part, by miR-155 levels. (B) In the context of ectopic expression of miR-155 in MDMs from multiple donors, curve estimation using a logarithmic regression model of miR-155 levels (independent variable) and relative infection data (dependent variable), indicated a good fit of the model (R2 = 0.514, p<0.01); however, careful evaluation of the data revealed the presence of an outlier that resulted in a moderate multiple correlation coefficient (R = 0.717). Removal of the outlier (circled in red) increased even further the goodness of the model (R2 = 0.804, p<0.001), suggesting that susceptibility to infection in miR-155-transfected MDMs is determined, in large part, by miR-155 levels. (TIF) [file ppat.1002937.s006.tif]

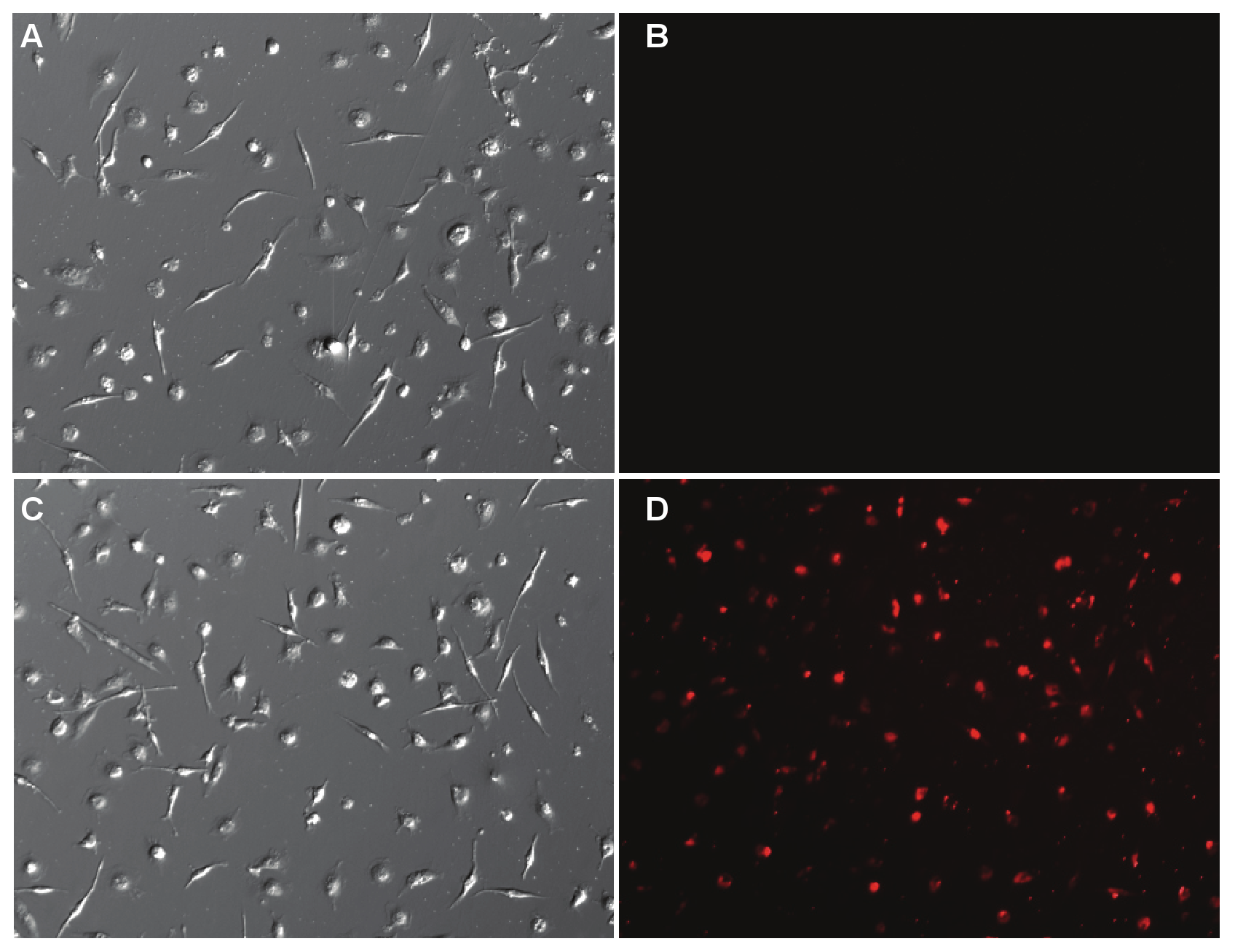

Supplement: Figure S7 — Efficiency of transfection of MDMs with an antagomir control. Primary human MDMs were untransfected (A,B), or transfected with the Ambion Cy3 dye-labeled Anti-miR scrambled negative control (anti-miR-scr, which does not target any known human miRNA) (Applied Biosystems), at an optimized concentration of 30 nM using Lipofectamine RNAiMax transfection reagent (Invitrogen) (C,D). At 24 h post-transfection, MDMs were washed with PBS and replaced with media, and bright field (A,C) and fluorescent (B,D) images (10× magnification) were obtained using an Olympus 1×81 deconvolution fluorescent microscope and SlideBook 5.0 software. The extent of Cy3 signal indicates a good transfection efficiency of primary MDMs. (TIF) [file ppat.1002937.s007.tif]

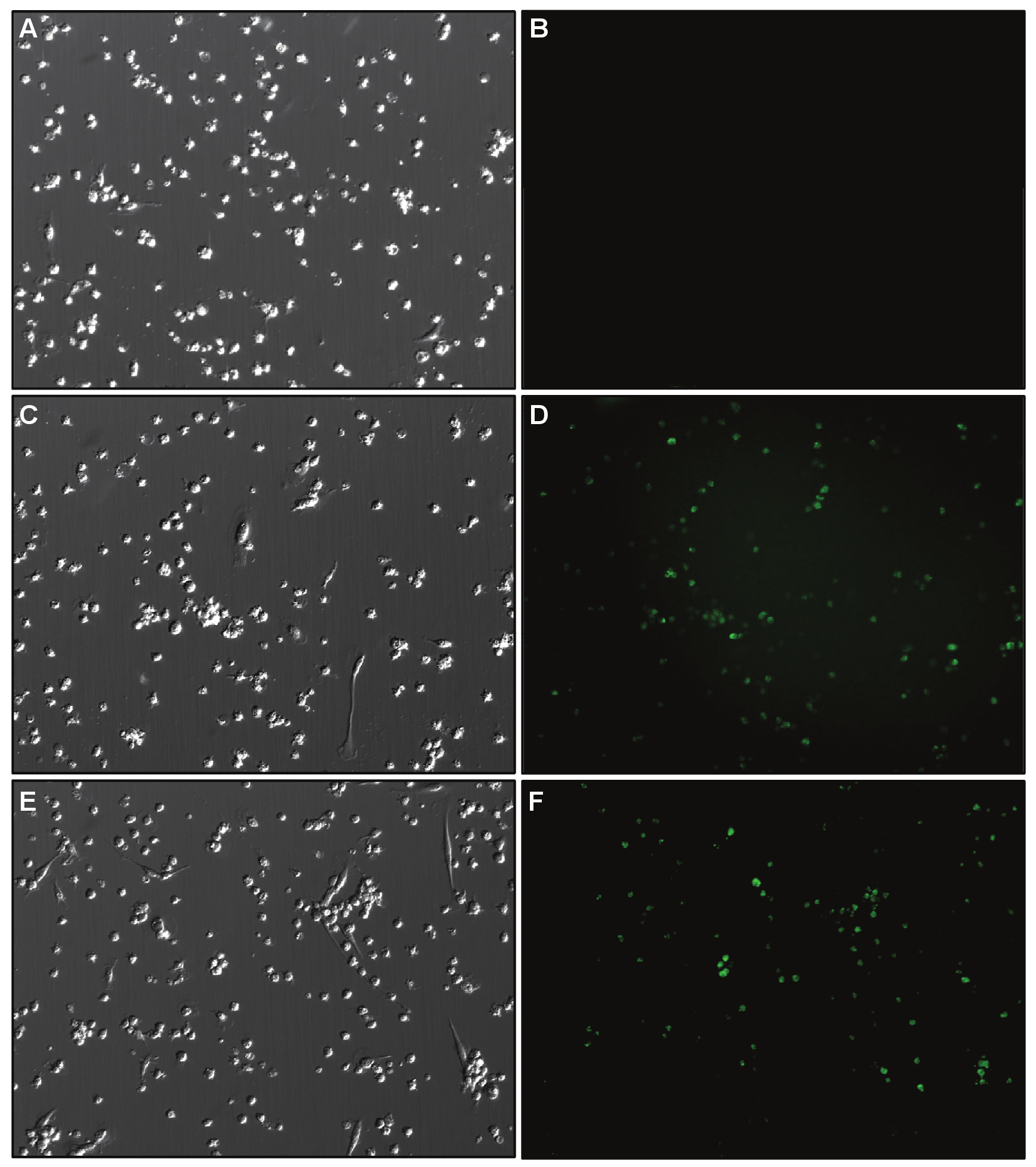

Supplement: Figure S8 — Efficiency of transfection of MDMs with plasmid DNA. Primary human MDMs were untransfected (A,B), or transfected with the GFP-encoding, miR-155 expression plasmid (pEZX-miR-155) (C,D) or scrambled control (pEZX-scrambled) (E,F) (GeneCopoeia), using jetPEI-Macrophage transfection reagent (Polyplus), following manufacturer's instructions. After 48 h, bright field (A,C,E) and fluorescent (B,D,F) images (10× magnification) were obtained using an Olympus 1×81 deconvolution fluorescent microscope and SlideBook 5.0 software (Intelligent Imaging Innovations, Inc.). The extent of GFP expression indicates a good transfection efficiency of primary MDMs. (TIF) [file ppat.1002937.s008.tif]

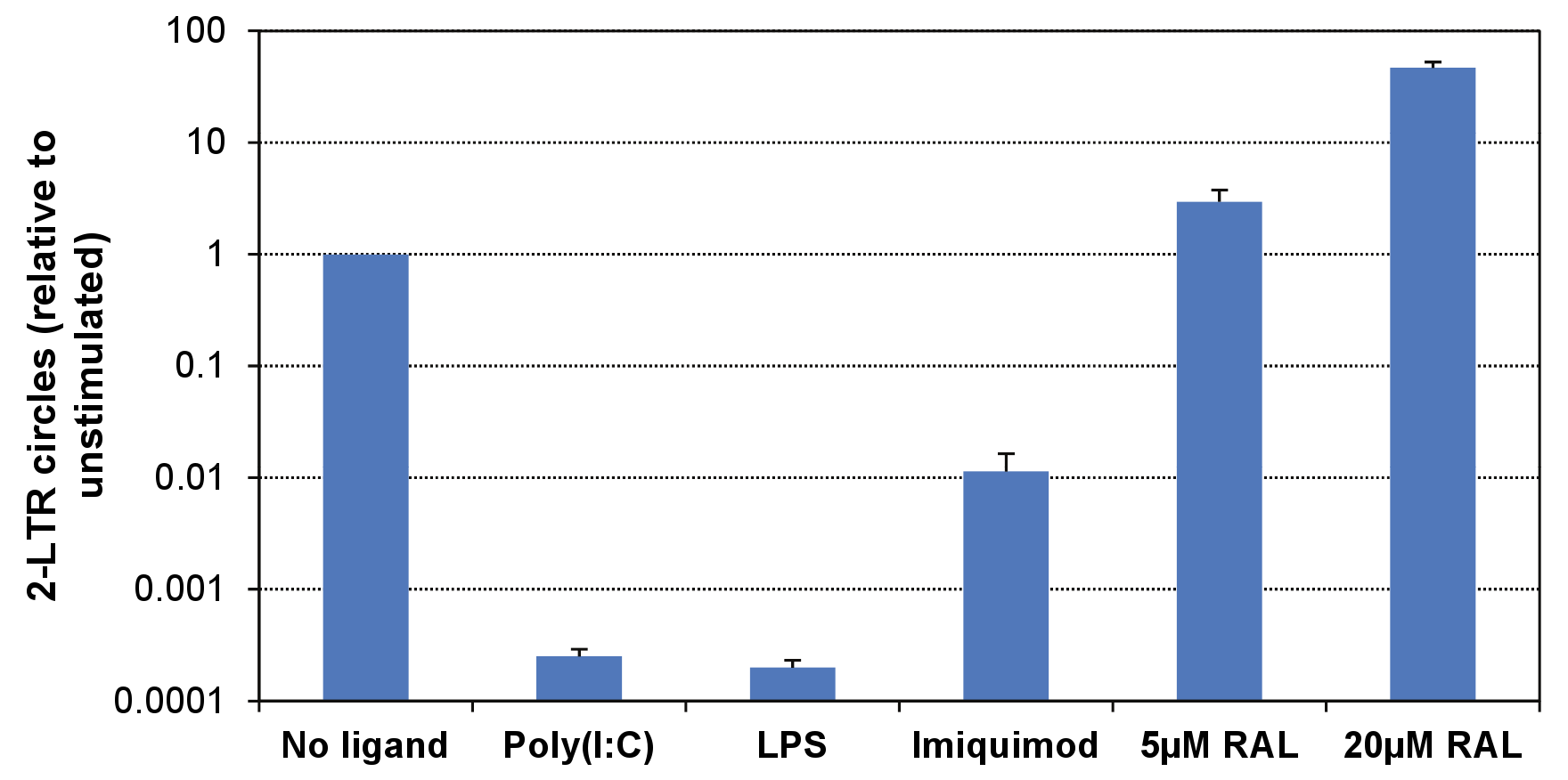

Supplement: Figure S9 — Detection of 2-LTR circles in MDMs. Total DNA was isolated at 48 h post-infection from BaL pseudotype-infected MDMs (shown from an individual donor) that were unstimulated, or stimulated with poly(I∶C), LPS or Imiquimod, or treated with two different concentrations of the integrase inhibitor Raltegravir (RAL). Real-time PCR was performed in total DNA for the relative quantitation of 2-LTR circles, as described in Materials and Methods. Results were calculated with respect to CCR5 (used as endogenous control to normalize for DNA recovery and number of cells), and are shown relative to the amounts detected in unstimulated cells, as mean ± SD from two independent quantitations. (TIF) [file ppat.1002937.s009.tif]
