# Supplementary material for: A Role for microRNA-155 Modulation in the Anti-HIV-1 Effects of Toll-Like Receptor 3 Stimulation in Macrophages
Source: PLoS Pathog. 2012 Sep 20;8(9):e1002937. doi: 10.1371/journal.ppat.1002937 (PMC3447756; doi:10.1371/journal.ppat.1002937)
Supplement: Table S1 — Primers and probes used for real-time PCR analysis of viral DNA products. (DOCX) [file ppat.1002937.s010.docx]

**Table S1. Primers and probes used for real-time PCR analysis of viral DNA products.**

| **Product** | **Oligo** | **Name** | **Sequence** |
| --- | --- | --- | --- |
| RU5 | Forward | hRU5-F2 | 5′ -GCCTCAATAAAGCTTGCCTTGA-3′ |
|  | Reverse | hRU5-R | 5′-TGACTAAAAGGGTCTGAGGGATCT-3′ |
|  | Probe | hRU5-P | 5′-FAM-AGAGTCACACAACAGACGGGCACACACTA-TAMRA-3′ |
| U5Ψ | Forward | MH531 | 5′-TGTGTGCCCGTCTGTTGTGT-3′ |
|  | Reverse | MH532 | 5′-GAGTCCTGCGTCGAGAGATC-3′ |
|  | Probe | LRT-P | 5′-FAM-CAGTGGCGCCCGAACAGGGA-TAMRA-3′ |
| 2-LTR | Forward | MH535 | 5’-AACTAGGGAACCCACTGCTTAAG-3’ |
|  | Reverse | MH536 | 5’-TCCACAGATCAAGGATATCTTGTC-3’ |
|  | Probe | MH603 | 5'-FAM-ACACTACTTGAAGCACTCAAGGCAAGCTTT-TAMRA-3' |
| ALU-LTR | Forward1 | Alu-f | 5’-GCCTCCCAAAGTGCTGGGATTACAG-3’ |
|  | Reverse1 | gag-rev | 5’-GCTCTCGCACCCATCTCTCTCC-3’ |
| ALU-LTR | Forward2 | LTR-f | 5’-GCCTCAATAAAGCTTGCCTTGA-3’ |
|  | Reverse2 | LTR-rev | 5’-TCCACACTGACTAAAAGGGTCTGA-3’ |
|  | Probe | LTRp^a^ | 5’-FAM-GTGCCCGTCTGTTGTGTGACTCTGGTAACTAG-TAMRA-3’ |
| PBGD | Forward | nPBGD-f | 5'-AGGGATTCACTCAGGCTCTTTCT-3' |
|  | Reverse | nPBGD-r | 5'-GCATGTTCAAGCTCCTTGGTAA-3' |
|  | Probe | nPBGD-pr | 5'-FAM-TCCGGCAGATTGGAGAGAAAAGCCTG-TAMRA-3' |
| CCR5 | Forward | CCR5-f | 5′-CCAGAAGAGCTGAGACATCCG-3′ |
|  | Reverse | CCR5-r | 5′-GCCAAGCAGCTGAGAGGTTACT-3′ |
|  | Probe | P-CCR5 | 5′-FAM-TCCCCTACAAGAAACTCTCCCCGG-TAMRA-3′ |

^a^Probe was shortened with respect to the published sequence.
