# Supplementary material for: A Role for microRNA-155 Modulation in the Anti-HIV-1 Effects of Toll-Like Receptor 3 Stimulation in Macrophages
Source: PLoS Pathog. 2012 Sep 20;8(9):e1002937. doi: 10.1371/journal.ppat.1002937 (PMC3447756; doi:10.1371/journal.ppat.1002937)
Supplement: Table S2 — Modulation of HIV-1 infection and miR-155 levels in MDMs with TLR stimulation (n = 10–12). (DOCX) [file ppat.1002937.s011.docx]

**Table S2. Modulation of HIV-1 infection and miR-155 levels in TLR-stimulated and unstimulated MDMs from multiple donors (n=10-12).**

| **Condition** | **Percent infection^a^ (range)** | **Fold change in miR-155^a^ (range)** |
| --- | --- | --- |
| Unstimulated | 100 | 1 |
| Poly(I:C) | 4.1 ± 2.2 (0.1 – 7.0) | 13.3 ± 6.1 (5.1 – 26.8) |
| LPS | 8.1 ± 5.1 (0.1 – 14.8) | 6.9 ± 3.5 (2.2 – 13.5) |
| Imiquimod | 51.9 ± 23.0 (23.0 – 87.0) | 2.0 ± 1.2 (0.8 – 4.4) |

^a^Infection and fold change are expressed as mean ± standard deviation.
