# Supplementary material for: A Role for microRNA-155 Modulation in the Anti-HIV-1 Effects of Toll-Like Receptor 3 Stimulation in Macrophages
Source: PLoS Pathog. 2012 Sep 20;8(9):e1002937. doi: 10.1371/journal.ppat.1002937 (PMC3447756; doi:10.1371/journal.ppat.1002937)
Supplement: Table S3 — Statistical analysis of miR-155 levels in TLR-stimulated and unstimulated MDMs. (DOCX) [file ppat.1002937.s012.docx]

**Table S3. Statistical analysis of miR-155 levels in TLR-stimulated and unstimulated MDMs.**

|  | **Poly(I:C)** | **LPS** | **Imiquimod** |
| --- | --- | --- | --- |
| Unstimulated | p < 0.001 | p < 0.001 | p < 0.05 |
| Poly(I:C) |  | p < 0.01 | p < 0.001 |
| LPS |  |  | p < 0.001 |

Statistical significance was calculated using a paired Student's t test.
